# Decreased Endothelin-1 bioavailability impairs aggressiveness of gallbladder cancer cells

**DOI:** 10.1186/s40659-025-00637-y

**Published:** 2025-08-20

**Authors:** Jetzabel Vidal-Vidal, David Brown-Brown, Nelson Quilaqueo-Millaqueo, Gaspar Peña-Münzenmayer, Carlos Spichiger, Claudia Quezada-Monrás, Flavio Salazar-Onfray, Julio C. Tapia, Ignacio Niechi

**Affiliations:** 1 Instituto de Bioquímica y Microbiología, Facultad de Ciencias, Universidad Austral de Chile, Valdivia, Chile; 2 Núcleo de Innovación en Biotecnología Apícola, Universidad Austral de Chile, NIBApi, Chile; 3 Millennium Institute on Immunology and Immunotherapy, Instituto de Bioquímica y Microbiología, Facultad de Ciencias, Universidad Austral de Chile, Valdivia, Chile; 4 Laboratorio de Biología Tumoral, Instituto de Bioquímica y Microbiología, Facultad de Ciencias, Universidad Austral de Chile, Valdivia, Chile; 5 Millennium Institute on Immunology and Immunotherapy, Faculty of Medicine, Universidad de Chile, Santiago, 8380453 Chile; 6 Disciplinary Program of Immunology, Institute of Biomedical Sciences, Faculty of Medicine, Universidad de Chile, Santiago, 8380453 Chile; 7 Laboratorio de Transformación Celular, Núcleo de Biología y Genética, Instituto de Ciencias Biomédicas, Facultad de Medicina, Universidad de Chile, Santiago, 8380453 Chile

## Abstract

**Background:**

Gallbladder cancer (GBC) is a highly lethal malignancy, often diagnosed at advanced stages when curative options are limited. Its rapid progression and high metastatic potential result in a 5-year survival rate below 5%. Recent evidence highlights the role of Endothelin-1 (ET1), a bioactive peptide, in promoting tumor aggressiveness through activation of its receptors (ETRs). However, therapeutic strategies have mainly focused on receptor inhibition, neglecting the modulation of ET1 availability. Therefore, this study aimed to evaluate the therapeutic potential of modulating ET1 levels through the application of recombinant Neprilysin (rNEP) to degrade ET1 or inhibition of Endothelin Converting Enzyme-1 (ECE1) to reduce its production in GBC cells.

**Methods:**

The effects of rNEP and the ECE1 inhibitor SM19712 were evaluated in GBC cell lines by assessing ET1 levels, nuclear β-catenin localization, transcript levels of target genes, and changes in proliferation, migration, invasion, and stemness-associated markers in vitro.

**Results:**

Both rNEP and SM19712 significantly reduced extracellular ET1 levels, nuclear β-catenin localization, and expression of genes such as CCND1, VEGFA, and BIRC5. Treatment also decreased the expression of EMT and stemness markers CD44 and Vimentin. Functionally, rNEP reduced cell migration, invasion, and colony formation, while SM19712 affected migration and colony formation. Isoform analysis revealed predominant expression of ECE1c, suggesting potential ET1-independent roles in invasion.

**Conclusion:**

Modulating ET1 bioavailability through enzymatic degradation or inhibition of its synthesis reduces aggressiveness in GBC cells. These findings support the use of rNEP and ECE1 inhibition as promising strategies for GBC treatment, although further in vivo validation is required.

**Supplementary Information:**

The online version contains supplementary material available at 10.1186/s40659-025-00637-y.

## Background

Gallbladder cancer (GBC) is recognized as the most prevalent and lethal form of biliary tract carcinoma [1]. Its 5-year survival rate fluctuates widely, ranging from 5 to 30%, depending on the disease detection, and unfortunately, most patients are diagnosed at an advanced stage, where the 5-year survival rate drops below 5% [2]. Incidence rates of GBC exhibit significant variation, with the highest reported cases annually in specific Eastern European countries, Asia, Bolivia, and Chile [1, 2]. Several risk factors contribute to the development of GBC, including gender, genetic-related geographic factors, chronic inflammation (cholecystitis), and gallstones (cholelithiasis) [1]. The incidence rates in certain regions may be attributed to the high prevalence of cholelithiasis, particularly in women, and genetic variants associated with the Mapuche ethnic group in South America. However, the exact association between these risk factors remains incomplete and unclear [2]. Despite these variations, chronic inflammation is believed to be a common factor, though the precise origin and development of the pathology remain unclear.

Recent research efforts have increasingly honed in on unraveling the molecular intricacies governing the aggressive behavior of different cancers related to chronic inflammation and tumor progression [3, 4]. Among the myriad factors, Neprilysin (NEP/CD10) and Endothelin-Converting Enzyme-1 (ECE1) have emerged as pivotal regulators, steering critical signaling pathways, specifically on Endothelin-1 (ET1) metabolism [5, 6]. ET1 signaling is mediated by two G protein-coupled receptors, ET_A_R and ET_B_R, leading to calcium mobilization and nuclear import of β-catenin and NF-κB [4, 7–11].

The ET1 signaling plays a crucial role in vascular homeostasis and has been implicated in the pathogenesis of various cancers, such as lung [8], ovarian [9], prostate [12], colon [13], pancreatic [14], and GBC [4]. Furthermore, genomic and transcriptomic studies of biliary tract carcinomas, including GBC, have revealed elevated expression of ET1 and its receptors (ETRs), which correlates with advanced tumor stages [15, 16]. Elevated levels of ET1 are observed in cancers with invasive phenotypes, which correlate with reduced survival, serving as a potential prognostic marker [17–19]. ET1 signaling has been implicated in the regulation of key oncogenic pathways, including β-catenin, which promotes epithelial-mesenchymal transition (EMT) [7]. EMT is closely linked to the acquisition of stemness properties, including self-renewal capacity, enhanced invasion, and therapy resistance, with markers such as CD44 and Vimentin commonly used to identify cells exhibiting stem-like and mesenchymal characteristics [4–18]. However, the role of ET1 metabolism, through its synthesis by ECE1 and degradation by NEP, in modulating EMT and stemness in GBC remains unexplored. ET1 has a plasma half-life of only 1 min, therefore, its pathophysiological effects depend on its continuous production by ECE1 and degradation by NEP, positioning these two proteins as potential targets to modulate the impact of ET1 in this cancer [5, 6].

NEP, a membrane-bound metalloendopeptidase, has garnered attention for its multifaceted roles in maintaining homeostasis of several peptides [20]. Of particular interest is NEP’s involvement in degradation of vasoactive peptides, including ET1. NEP exhibits a differential expression in various types of cancers; however, its levels are diminished in more aggressive tumor tissues [6, 20]. Therefore, NEP is believed to play a crucial role in the development and progression of cancer cells. Nevertheless, the role of this enzyme in GBC has not yet been described, making it a subject of study.

On the other hand, ECE1, a membrane-bound metallopeptidase key in the ET1 pathway, is responsible for the proteolytic processing of inactive Big-ET1 into its biologically active form ET1. Significantly, ECE1 exists as four isoforms (a, b, c and d), whose structures differ only in a small N-terminal cytosolic portion, while share a same extracellular domain responsible of its enzymatic activity [5]. The ECE1 isoforms display distinct tissue distributions, thus potentially contribute to distinct cellular responses [5]. The activity of ECE1 can be regulated by specific inhibitors, which target the extracellular enzymatic domain and prevent the conversion of Big-ET1 to ET1. One of such inhibitors, SM19712, is known to impede ECE1 activity and subsequently attenuate ET1 production [21, 22]. Of note, it has been demonstrated that isoform ECE1c enhances tumor aggressiveness in various tumor models [23–25], as well as its inhibition can reduce cell invasion [21]. There, increased ECE1c’s stability by phosphorylation is crucial [29], which promotes cancer stem cells traits related with proliferation, invasion, and chemoresistance [23–25].

Based on this, the aim of our study was to evaluate whether modulating ET1 bioavailability through ECE1 inhibition or the administration of recombinant NEP (rNEP) could reduce aggressive traits in gallbladder cancer cells, including EMT, stemness, migration, invasion, and tumorigenic potential.

## Materials and methods

### Cell culture and treatments

NOZ cell line is derived from GBC ascites metastasis [26] and CAVE1 cells were derived from a primary GBC tumor of a Chilean patient [27]. Cells were expanded in DMEM-HG medium supplemented with 10% FBS, 100 U/ml penicillin and 100 µg/ml streptomycin (Gibco) at 37 °C and 5% CO_2_, followed by storage in liquid nitrogen at -190 °C. Once a year, one nitrogen aliquot was thawed, expanded, and stored again at -80 °C. For experiments, one − 80 °C aliquot was thawed and grown in standard conditions. All experiments were performed within one year and cells were eliminated after 15 passages, as requested by each local biosecurity committee. Mycoplasma contamination was tested monthly with the EZ-PCR Mycoplasma Test kit (Biological Industries, Beit Haemek, Israel), being the last test performed six months ago and yielding no contamination. NEP (0.2 µg/ml) was purshased from Biolegend; and ET1 (100 nM) and SM191712 (20 µM for CAVE1 cells and 5 µM for NOZ) from Sigma Aldrich.

### Endothelin-1 quantification

Secreted ET1 was quantified using the ET1 Human ELISA Kit (Thermofisher, EIAET1). A cell density of 10^4^ cells/well were incubated for 48 h in serum free DMEM-HG. Cells were treated by 24 h with 0.2 µg/ml rNEP or SM191712 (5 µM in NOZ and 20 µM in CAVE1) ET1 levels were measured according to the manufacturer’s instructions and normalized to total protein content and cell number [4]. rNEP concentrations were selected based on preliminary dose-response experiments evaluating ET1 level modulation (data included in Fig. S1). In the case of CAVE1 cells, the concentration of SM19712 was selected because it was the dose at which changes in the analyzed markers were observed, considering that there is limited literature supporting the use of this drug at specific concentrations, particularly in GBC cell lines. Each experiment was performed with at least three biological replicates and three technical replicates to ensure reliability and reproducibility of the results.

### 3D-migration and invasion

Cells (5 × 10^4^ cells/chamber) were plated on the upper side of a polycarbonate Transwell chamber (6.5 mm, 8.0 μm, Corning, Lowell, MA, USA) for migration assay or in a 300 µg/mL matrigel-coated Transwell chamber for invasion assay. Cells were seeded in serum-free DMEM-HG with 10% FBS-DMEM-HG in bottom chamber as chemoattractant. Cells were treated with rNEP at 0.2 µg/mL, or with SM191712 at 20 µM for CAVE1 cells and 5 µM for NOZ cells. Cells were incubated at 37 °C for 4 h (migration) or 16 h (invasion). Cells in the top chamber were carefully removed with cotton swabs and cells that crossed through the chamber were fixed with 0.5% crystal violet solution in 10% Methanol for 10 min at room temperature. Cells were counted using the 10x objective in 5 different fields of the underside of the insert. The mean number of cells was normalized to 1 using the control condition and then plotted. Each experiment was performed with at least three biological replicates.

### RT-qPCR

Total RNA was extracted with TRIzol (Gibco) and quantified by NanoDrop. Reverse transcription was performed with 1 µg RNA plus M-MLV RT (Promega) following manufacturer instructions. qPCR was performed in a Stratagene MX30005P (Agilent Technologies Inc), using the ΔΔCt method and HPRT1 as a normalizer gene. For the reaction, buffer 2x Master mix qPCR Brilliant II Sybr^®^ Green (ThermoFisher, Waltham, MA, USA) was used, following the manufacturer’s instructions. The following genes were evaluated: *GAPDH*,* CCND1*,* VEGFA*,* BIRC5*,* CD44*,* Vimentin*,* ECE1a*,* ECE1b*,* ECE1c*,* and ECE1d.* Primers were published elsewhere [5–25]. Each experiment was performed with at least three biological replicates and three technical replicates to ensure reliability and reproducibility of the results. The control group was used as the reference to calculate fold changes.

### Western blot

Proteins (30–40 µg) were separated by SDS-PAGE (BioRad, Hercules, CA, USA), transferred to a 0.22 μm nitrocellulose membrane and then blocked with 5% non-fat milk in PBS-Tween 0.05%. Membranes were incubated at 4 °C overnight with primary antibodies followed by incubation for 1 h with a secondary HRP-conjugated anti-IgG antibody (Jackson Laboratories, 1:50,000 in 1X PBS-Tween 0.05%). Primary antibodies were, E-cadherin (CST #3195, 1:1000), β-actin (Santa Cruz Biotechnology #47778, 1:5000), ZEB1 (CST #3396, 1:1000), Lamin B1 (CST #12586, 1:1000), β-catenin (BD Biosciences #610153, 1:1000), NF-kB (CST #6956, 1:1000), GAPDH (Thermo-Fisher #MA5-15738, 1:1000), CD44 (CST #3570, 1:1000), Vimentin (CST #5741, 1:1000). Bands were revealed using the WestDura chemiluminescence system (Thermo-Fisher) and imaging was performed on a Syngene G: Box instrument (Synoptics, Cambridge, UK). Total proteins were normalized to β-actin, nuclear proteins to Lamin B1, and cytoplasmic proteins to GAPDH. The control group was used as the reference to calculate fold changes. Each experiment was performed with at least three biological replicates.

### Cell viability

CellTiter 96AQueous One Solution Cell Proliferation Assay (MTS) from Promega (Madison, WI, USA) was performed following manufacturer instructions. Briefly, 2.5 × 10^4^ cells were seeded in 96-well plates overnight and treated with NEP (0.2 µg/ml) or SM191712 (20 µM fot CAVE1 and 5 µM for NOZ) for 24 h. Cells were incubated with MTS reagent for 2 h and absorbance was measured at 490 nm using a microplate reader (Synergy HT, BioTek Instruments, Inc.). Each experiment was performed with at least three biological replicates and three technical replicates to ensure reliability and reproducibility of the results.

### Colony formation

In a 6-well plate, 1000 cells per well were seeded in DMEM supplemented with 10% FBS. Cells were treated with rNEP at 0.2 µg/mL, or with SM191712 at 20 µM for CAVE1 cells and 5 µM for NOZ cells. The medium and treatments were refreshed every 48 h, and the cells were grown for 7 days. Afterward, the culture medium was removed, and the wells were washed twice with PBS. A 0.5% crystal violet solution in 25% methanol (500 µL) was added to each well and incubated for 10 min at room temperature. The crystal violet was then removed, and the wells were gently washed with dH_2_O under constant agitation. One optical field was photographed, colonies were manually counted, and the fold change compared to the untreated control was plotted. Each experiment was performed with at least three biological replicates.

### Calcium release

Cells were loaded with 0,5 ng/ml Fluo-4 AM dye, 10 min at 37 °C and incubated with 100 nM ET1 (stimulation at 50 s) or in combination with 1 µM Ambrisentan (AMB) from 0 to 1100 s. Imaging was performed using an inverted microscope (Nikon Eclipse TE300) equipped with a Lambda 10 − 2 Filter Wheel Imaging System coupled to a digital camera (Retiga R1, QImaging). A plan fluor 40X, NA 0.75, objective was used. Images were acquired by excitation at 494 nm and emission captured at 506 nm using Micro-Manager 1.4 microscopy software. The ratio of fluorescence (F) to initial fluorescence (F₀) was plotted as a function of 5-second time intervals.

### Statistical analysis

Statistical analysis and graphical representations were conducted using GraphPad Prism 8.1 software. Values were presented as mean ± SD from a minimum of three independent experiments. Statistical analysis was performed on normalized data using the unpaired t-Student test for unpaired data and one-way ANOVA for data groups.

## Results

### ET1 bioavailability is diminished by Neprilysin and ECE1 Inhibition in GBC cells

**Fig. 1:**
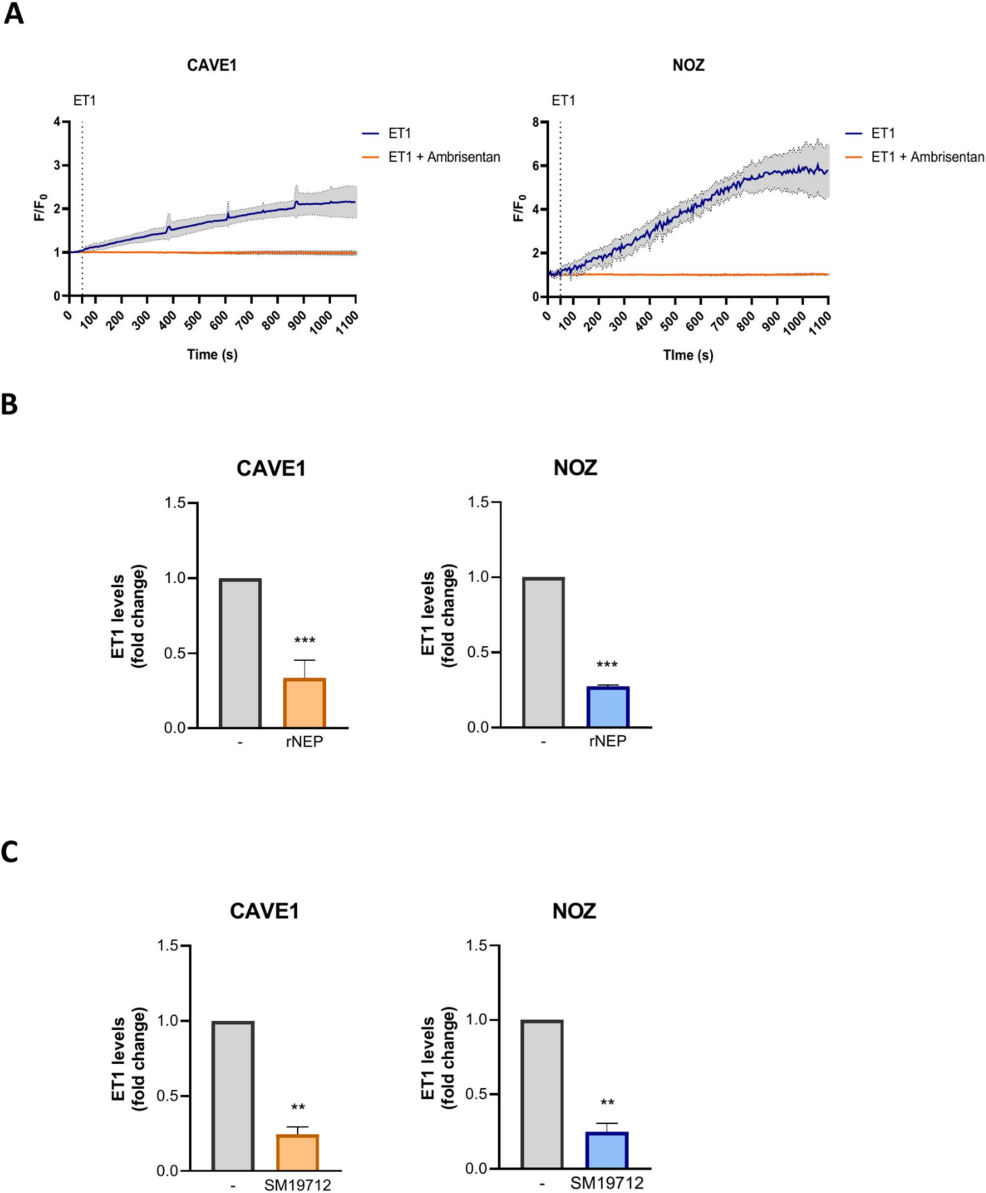
ET1 triggers calcium release, which extracellular levels can be reduced by recombinant NEP or ECE1 inhibition. (**A**) Calcium release assay in response to ET1 stimulation. CAVE1 and NOZ cells were loaded with Fluo-4 AM and exposed to ET1, with and without the ET_A_R antagonist Ambrisentan (AMB). Calcium release was measured over time using fluorescence microscopy. (**B**) CAVE1 and NOZ cells were treated with 0.2 µg/ml rNEP. The plot shows extracellular ET1 levels quantified by ELISA in the culture medium. (**C**) Same as in B with ECE1 inhibitor SM19712 (5 µM in NOZ and 20 µM in CAVE1). Data represent averages ± SD (*n* = 3). ANOVA and Student’s tests were used. *p < 0.05 **p < 0.01

Our group demonstrated for the first time that aggressive features of GBC cells could be mitigated by blocking ET1 receptors ET_A_R and ET_B_R with Macitentan, a dual antagonist [4]. We first assessed whether GBC cells respond to stimulation with ET1, and whether NEP-dependent degradation exerts similar effects on tumor aggressiveness. To demonstrate the susceptibility of cells to ET1, a calcium release measurement assay was conducted, revealing that our both GBC models, NOZ cell line and CAVE1 primary culture, effectively responded to ET1, which was also counteracted with the selective ET_A_R antagonist Ambrisentan (Fig. 1A).

Therefore, we aimed to assess how ET1 production and degradation impact the aggressive phenotype of GBC cells. Considering that ECE1 is expressed as four variants and that isoform ECE1c has been shown to promote aggressiveness traits in other cancers [23–25], mRNA levels of these isoforms were analyzed by RT-qPCR. As observed in Fig. S2, the most expressed isoform was ECE1c, while others were the least represented, in our both models. Further, extracellular ET1 levels were assessed upon treatment of both GBC cells with either recombinant neprilysin (rNEP), which degrades ET1, or the ECE1 inhibitor SM19712, which decreases ET1 activation. As expected, the results showed a significant decrease in extracellular ET1 levels in both GBC models with either rNEP (Fig. 1B) or SM19712 (Fig. 1C).

### Neprilysin and ECE1 inhibition led to reduced markers associated to ET1 signaling in GBC cells

**Fig. 2:**
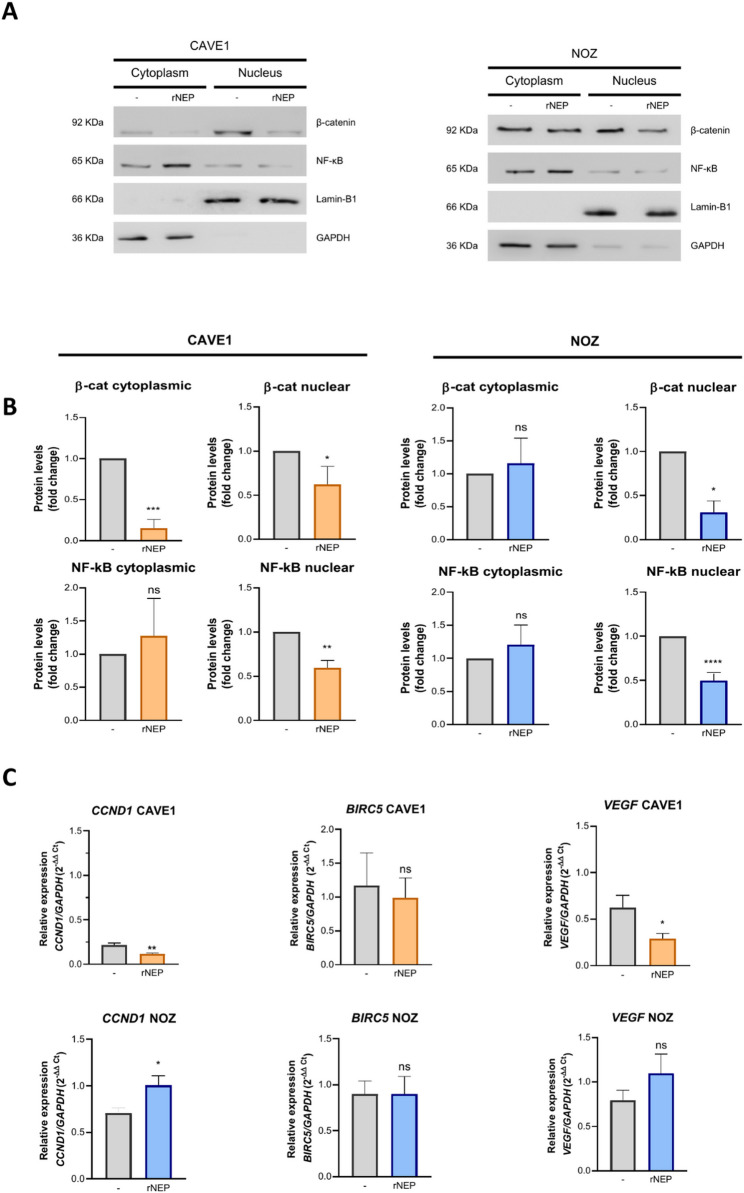
rNEP decreases ET1 signaling markers in GBC cells. (**A**) CAVE1 and NOZ cells were treated with 0.2 µg/ml recombinant Neprilysin (rNEP), and nuclear and cytoplasmic protein levels of β-catenin and NF-κB were assessed by Western blot in of CAVE1 and NOZ cells. Lamin B1 and GAPDH were used as markers for nuclear and cytoplasmic fractions, respectively. Representative images from three independent experiments are shown. (**B**) Quantification of protein expression levels from Western blots shown in A. (**C**) ET1 pathway target gene expression changes in response to rNEP treatment were measured by RT-qPCR in CAVE1 and NOZ cells. Data represent averages ± SD (*n* = 3). ANOVA and Student’s tests were used. *p < 0.05 **p < 0.01

We aimed to assess whether reduction of extracellular ET1 could diminish the expression of markers associated with activation of the ET1 pathway, as NF-κB and β-catenin nuclear translocation [4]. As observed, rNEP decreased nuclear levels of both, NF-κB and β-catenin (Fig. 2A), as well as target genes of the canonical Wnt pathway (Fig. 2C), highlighting a significant effect on *CCND1* (cyclin-D1) and *VEGF* mRNA levels, with no changes in *BIRC5* (survivin), but interestingly in only CAVE1 cells. These results suggest that treatment with rNEP may impact expression of key proteins related with a ET1-dependent activation of the canonical Wnt pathway.

**Fig. 3:**
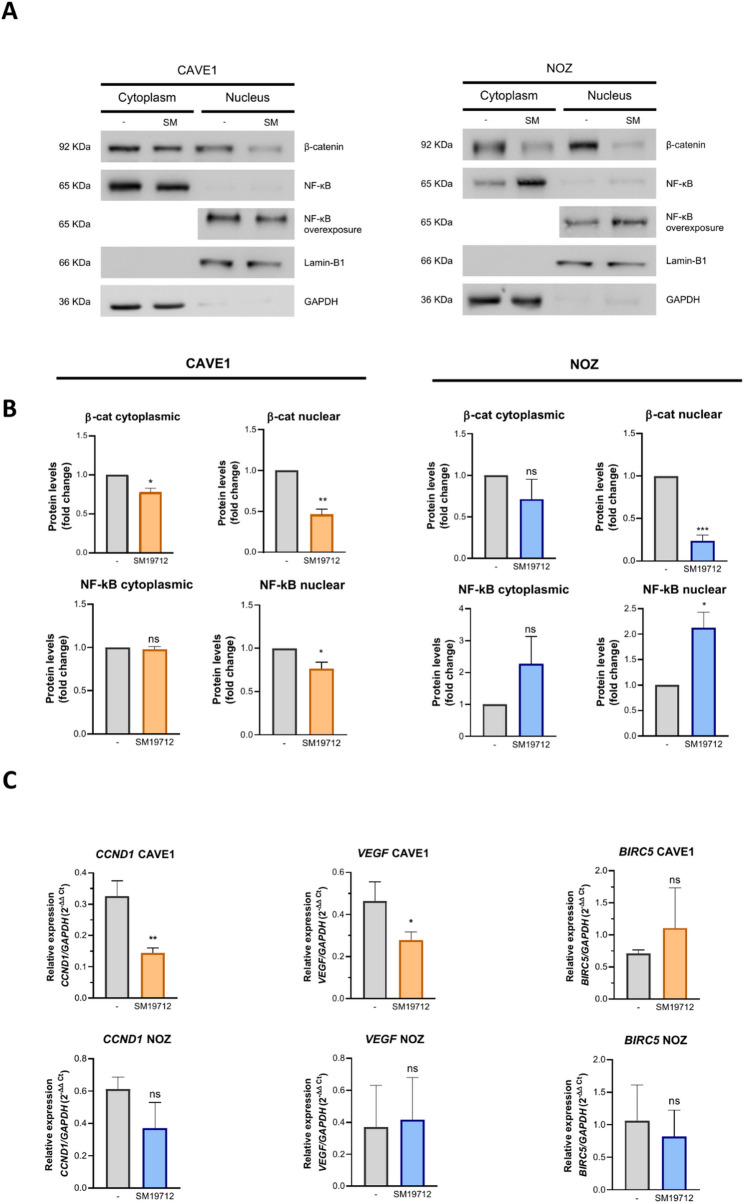
ECE1 inhibition by SM19712 decreases ET1 signaling markers in GBC cells. **A**. CAVE1 and NOZ cells were treated with the ECE1 inhibitor SM19712 (5 µM in NOZ and 20 µM in CAVE1), and nuclear and cytoplasmic protein levels of β-catenin and NF-κB were assessed by Western blot. Lamin B1 and GAPDH were used as markers for nuclear and cytoplasmic fractions, respectively. Representative images from three independent experiments are shown. (**B**) Quantification of protein expression levels from Western blots shown in A. (**C**) ET1 pathway target gene expression changes in response to SM19712 treatment were measured by RT-qPCR in CAVE1 and NOZ cells. Data represent averages ± SD (*n* = 3). ANOVA and Student’s tests were used. *p < 0.05 **p < 0.01

Considering the short half-life of ET1 and its effects being dependent on its production by ECE1, we investigated whether inhibition of this enzyme may produce effects similar to ET1 depletion by rNEP. As expected, in both GBC models reduced extracellular ET1 levels upon inhibition of ECE1 with SM19712 led to decreased nuclear NF-κB and β-catenin presence (Fig. 3A), which correlated with significant diminished mRNA levels of canonical Wnt target genes *CCND1* and *VEGF* (Fig. 3C) although, very surprisingly, also in only CAVE1 cells. Altogether, these results demonstrate that decreasing ET1 levels, either through inhibition of its production or enhancement of its degradation, reduces aggressive characteristics in GBC cells.

### Reduction of ET1 bioavailability decreases expression of mesenchymal and stemness markers

**Fig. 4:**
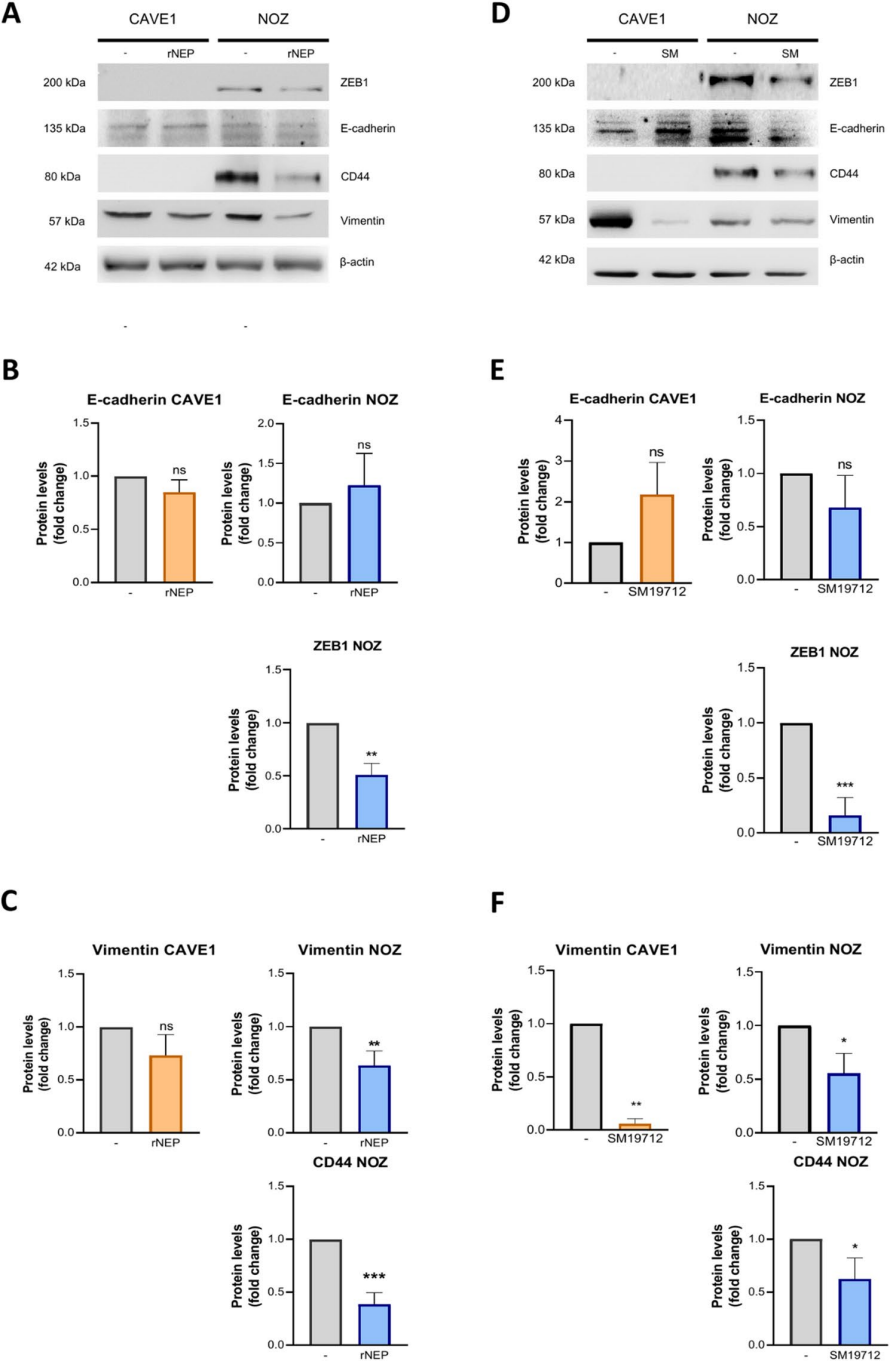
rNEP and SM19712 reduce cell migration and alter EMT-related protein expression. **A.** Expression of EMT-related proteins (E-cadherin and ZEB) and stem-associated markers (CD44 and Vimentin) was assessed by Western blot in NOZ and CAVE1 cells treated with 0.2 µg/ml recombinant NEP (rNEP), using β-actin as a loading control. **B-C.** Quantification of protein expression levels from Western blots shown in A. **D.** Expression of EMT-related proteins and stem-associated markers was assessed by Western blot in cells treated with the ECE1 inhibitor SM19712 (5 µM in NOZ and 20 µM in CAVE1), using β-actin as a loading control. **E-F.** Quantification of protein expression levels from Western blots shown in D. ANOVA and Student’s tests were used. *p < 0.05 **p < 0.01

There is no evidence of effects of ECE1 activity on migration in GBC cells, as well as literature is scarce in reports on the effect of NEP on this process. Firstly, we found that treatment with rNEP or SM19712 differentially modulated the levels of several proteins related to EMT and stemness. Thus, treatment with rNEP significantly diminished protein levels of the transcription factor ZEB1 only in NOZ cells (absent in CAVE1 cells), however, the same treatment had no effect on levels of the known down-regulated epithelial marker E-cadherin in both GBC models (Fig. 4A). Conversely, rNEP treatment did have a significant effect on the mesenchymal marker vimentin, whose protein levels decreased in both GBC models (Fig. 4A). Like occurred with ZEB1 in CAVE1 cells, protein levels of the stemness marker CD44 was absent in these cells, being significantly diminished upon treatment with rNEP (Fig. 4A). Noteworthy, the ECE1 inhibitor SM19712 showed an extraordinarily similar effect compared to rNEP on protein levels of ZEB1, E-cadherin, vimentin and CD44 in both GBC models (Fig. 4D). Together, these results showed that reducing ET1 levels significantly impact expression of ZEB1 and CD44 in GBC cells.

### Neprilysin and ECE1 inhibitor impair cell migration and invasion of GBC cells

**Fig. 5:**
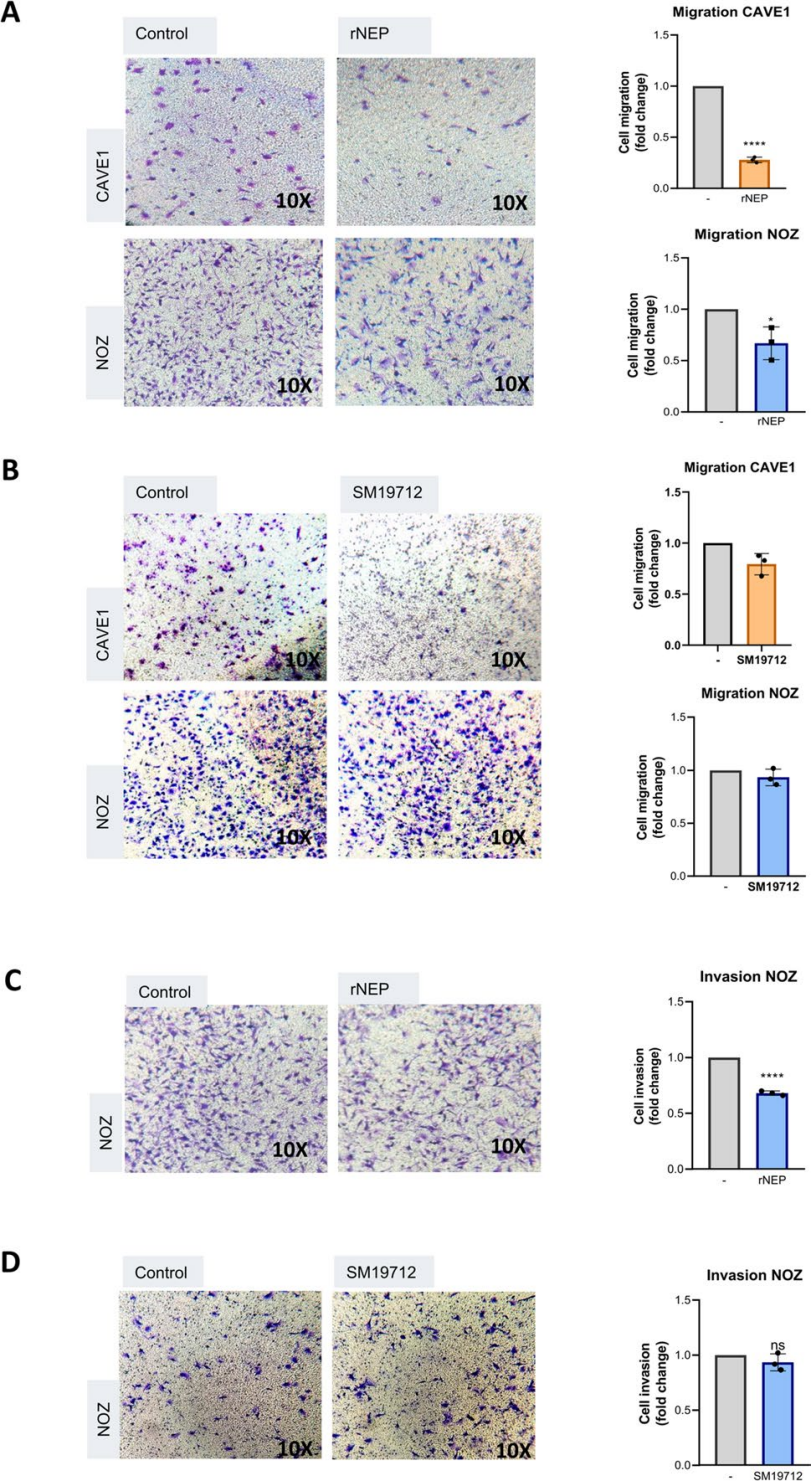
rNEP reduces cell migration and invasion in GBC cells. (**A**) CAVE1 and NOZ cells were seeded in a transwell chamber in FBS free medium with 10% FBS in the lower bottom as a chemoattractant. Cells were incubated at 37 °C for 4 h in presence or absence of 0.2 µg/ml rNEP. Cells were fixed and stained with crystal violet and counted using the 10X objective in 5 different fields. Control was normalized to 1 and finally plotted as fold changes. Scale bar: 100 nm. Data represent averages ± SDs (*n* = 3). (**B**) Same as in A the ECE1 inhibitor SM19712 (5 µM in NOZ and 20 µM in CAVE1). (**C**) NOZ cells were seeded in a matrigel-coated transwell chamber in FBS free medium with 10% FBS in the lower bottom as a chemoattractant. Cells were incubated at 37 °C for 16 h in presence or absence of 0.2 µg/ml rNEP. Cells were fixed, stained and plotted as in A. (**D**) Matrigel-coated transwell as in C, with 5 µM SM19712. Scale bar: 100 nm. Data represent averages ± SEMs (*n* = 3). ANOVA and Student’s tests were used. *p < 0.05 **p < 0.01

In agreement with the levels of ZEB1 and vimentin proteins, rNEP and SM19712 treatments significantly decreased the migratory capacity of both GBC models (Fig. 5A, B). Because CAVE1 cells are known not exhibiting invasive capability [4], we used only NOZ cells to study their invasive capability due to its metastatic origin and elevated migration capability. Thus, our results showed that treatment with rNEP, but not SM19712, led to a significant decrease of invasive capability in NOZ cells (Fig. 5C-D). Of note, a MTS assay was performed in both GBC models for the same treatments, but no changes were observed, indicating that our findings are due to cell migratory/invasive changes but not cell death (Fig. S3). Thus, our results show that a reduced ZEB1 and vimentin expression upon impairing ET1 bioavailability significantly affect cell migration and invasion of GBC cells.

### Colony formation is decreased upon ET1 reduction in GBC cells

**Fig. 6:**
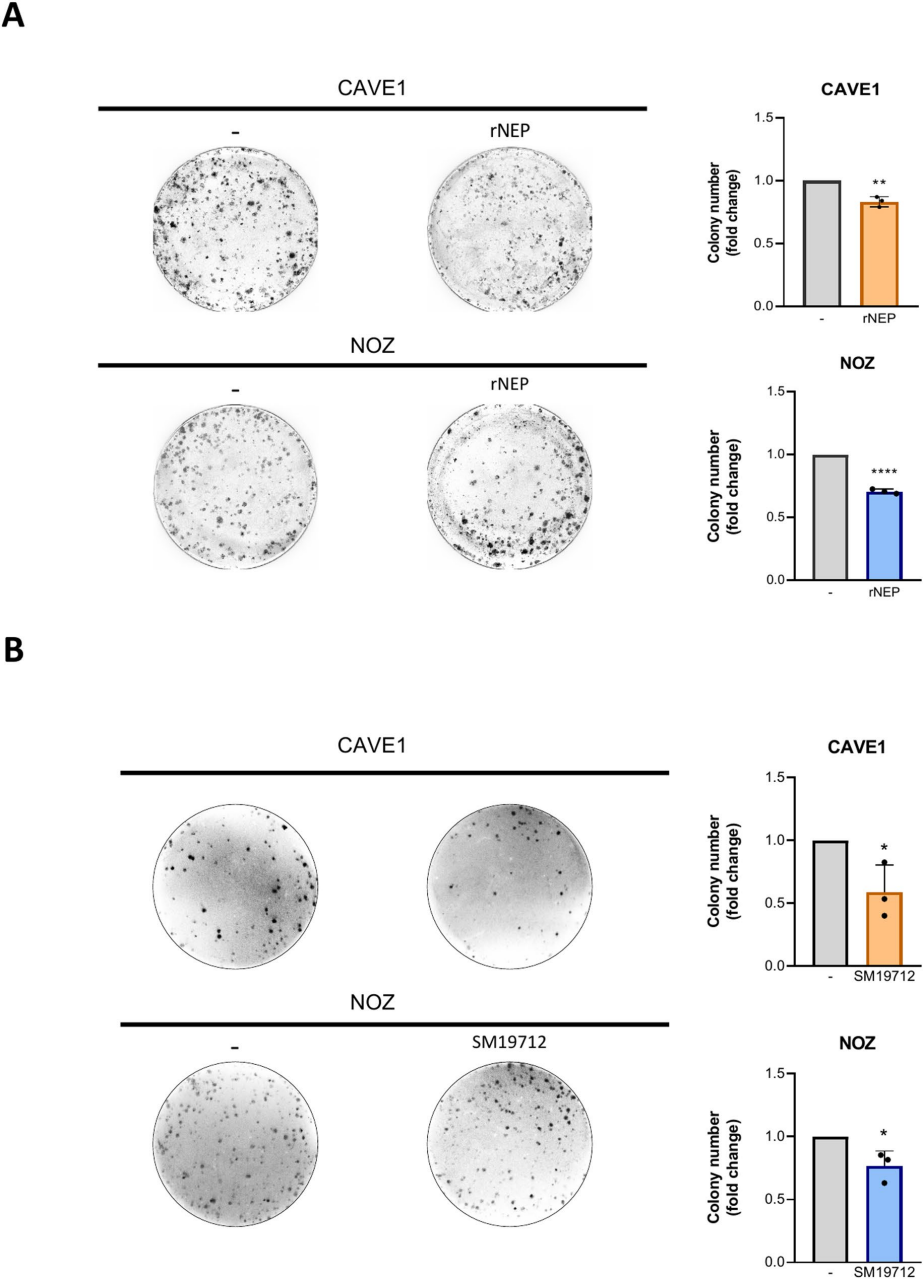
rNEP and SM19712 inhibit colony formation and reduce stem-associated markers. **A**. CAVE1 and NOZ were seeded in 6-well plates and treated with 0.2 µg/ml rNEP or ECE1 inhibitor SM19712 (5 µM in NOZ and 20 µM in CAVE1) every 24 h for 7 days. After incubation, cells were stained with crystal violet and colonies were quantified and plotted. Representative images from three independent experiments are shown. Statistical analysis was conducted using the Student’s t-test (*= *p* < 0.05, ** = *p* < 0.01; **** = *p* < 0.0001)

Recombinant neprilysin and ECE1 inhibition led to decreased protein levels of vimentin and CD44, which are also associated to acquired stemness traits in several types of cancer. Thus, in an approach to study an important stemness trait of our GBC models, the in vitro tumorigenic capability was evaluated using a colony formation assay. As expected, increased degradation of ET1 with rNEP (Fig. 6A) and inhibition of ECE1 with SM19712 (Fig. 6B) led to a significant decrease of colony formation in both CAVE1 and NOZ cells. Altogether, these results demonstrated that reducing ET1 bioavailability significantly decreased the tumorigenic capability of GBC cells, underscoring the key role of ET1 metabolism in this type of cancer.

## Discussion

**Fig. 7:**
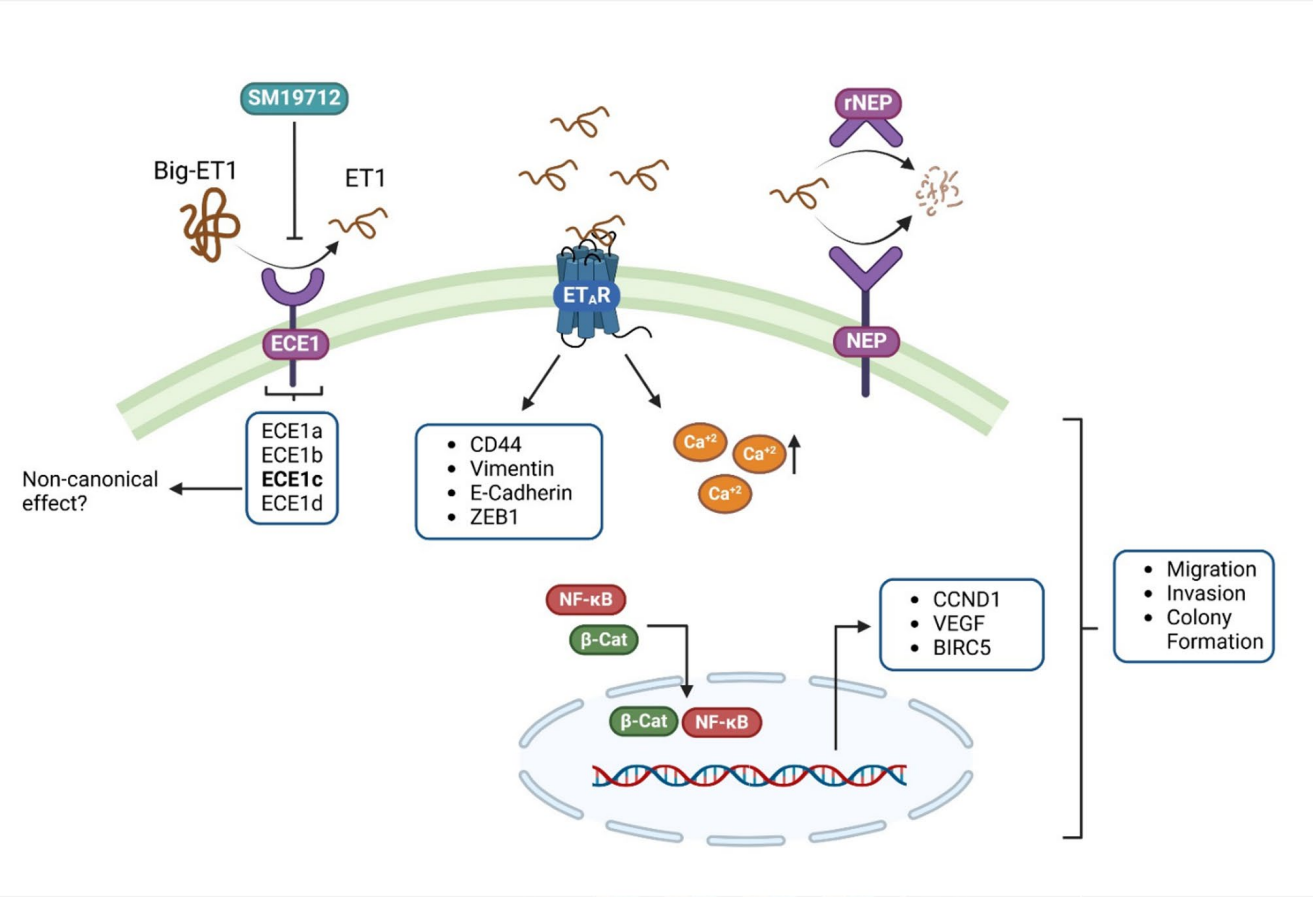
Targeting ET1 metabolism significantly impairs aggressiveness of GBC cells. rNEP and ECE1 inhibition both reduce extracellular ET1 levels, impair cell migration and invasion, and decrease colony formation. Additionally, rNEP affects stemness-related markers, while ECE1c is identified as the predominant isoform in GBC cells, potentially contributing to invasive behavior. The schematic provides a visual overview of the experimental approaches and outcomes, emphasizing that targeting ET1 metabolism could represent a promising therapeutic strategy for further investigation in preclinical models

Advanced tumor stages at which patients with GBC are diagnosed leads to metastasis al low 5-year survival rates, rendering the subsequent application of adjuvant therapies less effective due to the cancer’s aggressiveness. In this study, we proposed that the application of rNEP or the inhibition of ECE1 could be a good option as a therapeutic tool to mitigate tumor aggressiveness by degrading ET1 or inhibiting its production, respectively (Fig. 7).

### ET1 extracellular levels in endogenous ECE1 and NEP regulation

As a preliminary approach to the effect of the application of rNEP led to an increase in endogenous NEP protein levels. Interestingly, endogenous NEP not only has functions associated with its catalytic activity but also functionality attributed to its intracellular domain. It has been observed to interact with different proteins, such as PTEN and Lyn, impairing PI3K/AKT activation and preventing the phosphorylation of FAK thereby reducing cell migration processes [20, 28]. The application of rNEP also resulted in a decrease in protein levels of ECE1 and a common element in the post-translational regulation of both enzymes is the protein kinase CK2, which phosphorylates the intracellular domain of both ECE1 and NEP [29, 30]. Phosphorylation of ECE1c by CK2 stabilizes its protein levels thereby enhancing aggressiveness in glioblastoma, lung and colorectal cancer cells [21, 25, 29]. On the other hand, CK2-mediated phosphorylation of NEP leads to the loss of interaction with PTEN preventing the dephosphorylation of PIP3 and enhancing AKT activation, which is widely associated with cell survival, differentiation, and proliferation in gallbladder cancer [30, 31]. It should be noted that the presence of NEP has been analyzed in tumor samples of GBC, however, the results are not yet conclusive enough to classify it as a tumor marker by itself [32]. In conclusion, the effects observed in the reduction of characteristics associated with tumor progression may not be exclusive to its catalytic activity, but may also be indirectly mediated by the modulation of the endogenous function of ECE1 and NEP themselves.

### rNEP and ECE1 inhibition impair ET1 signaling activation

It was previously demonstrated that ET1 promotes the stability and co-transcriptional activity of β-catenin, and the blockage of its receptors with Macitentan, decrease β-catenin nuclear localization [4]. These previous results suggest that rNEP and SM19712 may be affecting β-catenin function indirectly through the reduction of ET1 extracellular levels and the subsequently ETRs inactivation. In fact, similar to Macitentan, rNEP and SM19712 were able to decrease nuclear β-catenin levels, which could potentially be linked to the reduction of its role as a transcriptional co-factor. The results obtained for the target genes *CCND1*, *VEGF*, and *BIRC5* reveal a clear variation between the CAVE1 and NOZ cell lines. In CAVE1, a decrease in the transcript levels of *CCND1* and *VEGF* was observed, which aligns with the reduction in nuclear β-catenin, a key cofactor regulator of these genes. However, this relationship remains inconclusive as their transcriptional activity was not directly assessed. It is important to note that CAVE1 is a less invasive but highly proliferative cell line, which may explain its increased susceptibility to the negative regulation by ET1. Interestingly, the NOZ cell line did not show decreased mRNA levels following treatments, which could be related to the lower proliferative index of this cell line compared to CAVE1, despite the reduction in nuclear β-catenin.

### rNEP and ECE1 inhibition in stemness traits

An important feature in the diagnosis and prognosis of GBC is the tissue de-differentiation which is strongly related to cell stemness, a key process for maintaining and forming new tumors, such as CD44 and Vimentin [33, 34]. In our study, both rNEP and SM19712 decreased the levels of stem-associated markers which are related to the acquisition of stem-like characteristics, such as invasion, self-renewal, migration, and angiogenesis, which are crucial for the maintenance and spread of GBC cells to other tissues [34]. In this regard, a decrease in migratory, invasive, and colony formation capacity was observed in cells treated with rNEP. In the case of ECE1 inhibition, a decrease in migration and colony formation was also observed, but no changes were observed in cell invasion.

Studies have shown that rNEP is effective in various conditions, including Alzheimer’s disease, where reduced NEP, a key protein in degrading beta-amyloid peptide, leads to amyloid plaque formation [35]. Additionally, rNEP-loaded collagen hydrogels have been used to cross the blood-brain barrier and degrade amyloid plaques in a transgenic Alzheimer’s mouse model [36]. Furthermore, decreased NEP levels are associated with inflammatory reactions and pulmonary fibrosis caused by viral infections in the lungs. Given its role in the renin-angiotensin system, converting angiotensin I into the protective angiotensin-(1–7), NEP is suggested as a potential therapeutic agent for such conditions [37]. This indicates that our strategy using rNEP has potential for clinical application.

### ECE1 role in cell invasion and a possible ET1-independent mechanism

Despite invasion markers decreased under treatment with ECE1 inhibitor SM19712, this was not reflected in the regulation of the invasive capacity. This result is quite surprising, as suggested in other models, ECE1 could have a role in invasion independent of catalytic activity and ET1 production [23, 38]. In fact, it has been suggested that the independent role may be given by differences between its isoforms, which have the same activity but have small structural differences in their cytoplasmic region, suggesting an ET1-independent or non-canonical role [38–40]. Specifically, the ECE1c isoform has been associated with cellular invasion not mediated by ET1 [23, 24, 38, 40]. To address the potential non-canonical role of the isoforms, we first assessed the relative expression of transcripts from the four isoforms. While this had previously been evaluated in non-tumor models using conventional PCR techniques [41], our group recently demonstrated the feasibility of quantitatively evaluating these isoforms in human non-small cell lung cancer (NSCLC) cells using RT-qPCR [25]. In that study, we observed that NSCLC cell lines predominantly expressed ECE1c, a finding that aligns with the results we now present in gallbladder cancer (GBC). Our results strongly demonstrate that isoform c is the most represented in GBC, suggesting that inhibition of ECE1 activity alone is insufficient to prevent its pathological effects. This is likely due to the non-canonical role described for this isoform. Furthermore, it is important to note that NOZ has twice ECE1c mRNA levels compared to the CAVE1 primary culture, which could suggest that elevated levels of this isoform promote the acquisition of an invasive phenotype. Altogether, our results suggest that ET1 metabolism could be a potential therapeutic target for GBC, although there are limitations due to the use of in vitro data and the lack of animal or patient validation. Further investigation is needed to understand the specific mechanisms of these enzymes, particularly those independent of their catalytic activities, in preclinical models.

## Supplementary Information

Below is the link to the electronic supplementary material.

**Supplementary Material 1**: **Figure Supplementary 1.** GBC cells were treated with rNEP or SM19712 at different concentrations at 24h. The plot shows extracellular ET1 levels quantified by ELISA in the culture medium. Data represent averages ± SEM (*n* = 3) **** = *p* < 0.0001.

**Supplementary Material 2**: **Figure Supplementary 2**. ECE1c is the predominant isoform in GBC cells. The expression profile of the ECE1 mRNA isoforms was determined by the 2^−Δct^ method using the *HPRT1* gene as the normalizer in both cell models and a common calibrator. The relative expression levels of total ECE1 (A) and ECE1c (B) were compared between both cell models. The relative expression levels of mRNA of the four isoforms (a-d) were compared in NOZ cells (C) and in CAVE1 cells (D). Statistical analysis was conducted using the Anova (*= *p* < 0.05, ** = *p* < 0.01; **** = *p* < 0.0001).

**Supplementary Material 3**: **Figure Supplementary 3.** Cells were seeded in 96-well plates for 24h and treated with 0.2µg/ml rNEP or ECE1 inhibitor SM19712 (5 µM in NOZ and 20 µM in CAVE1) for 72h. Cells were incubated with MTS reagent for 2h and absorbance was measured at 490nm and plotted as percentage. Data represent averages ± SEMs (*n* = 3). ANOVA and Student’s tests were used. *p < 0.05 **p < 0.01.

**Supplementary Material 4**

## Data Availability

Not applicable.
