# Supplementary figures and images for: Decreased Endothelin-1 bioavailability impairs aggressiveness of gallbladder cancer cells
Source: Biol Res. 2025 Aug 20;58:57. doi: 10.1186/s40659-025-00637-y (PMC12366186; doi:10.1186/s40659-025-00637-y)

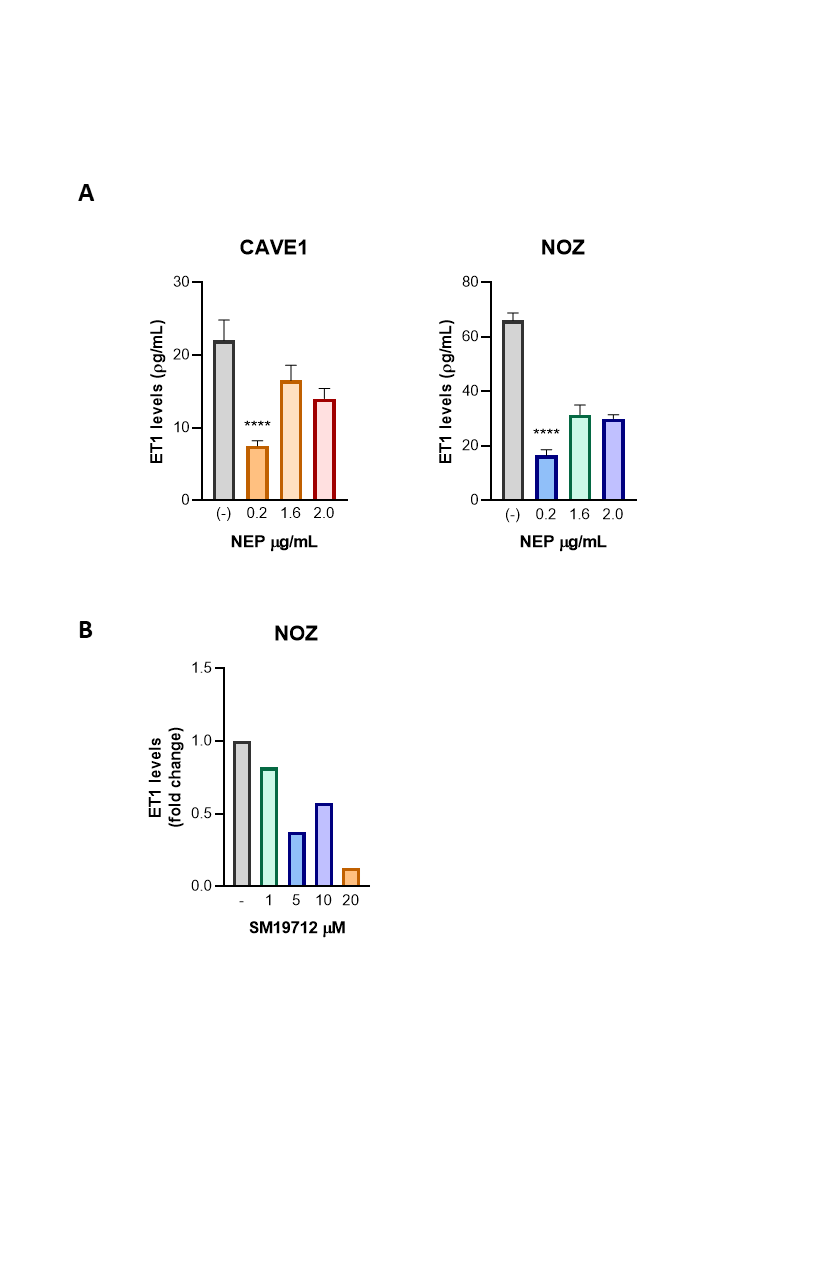

Supplement: Supplementary file 1 — Supplementary Material 1: Figure Supplementary 1. GBC cells were treated with rNEP or SM19712 at different concentrations at 24h. The plot shows extracellular ET1 levels quantified by ELISA in the culture medium. Data represent averages ± SEM (n = 3) **** = p < 0.0001. [file 40659_2025_637_MOESM1_ESM.png]

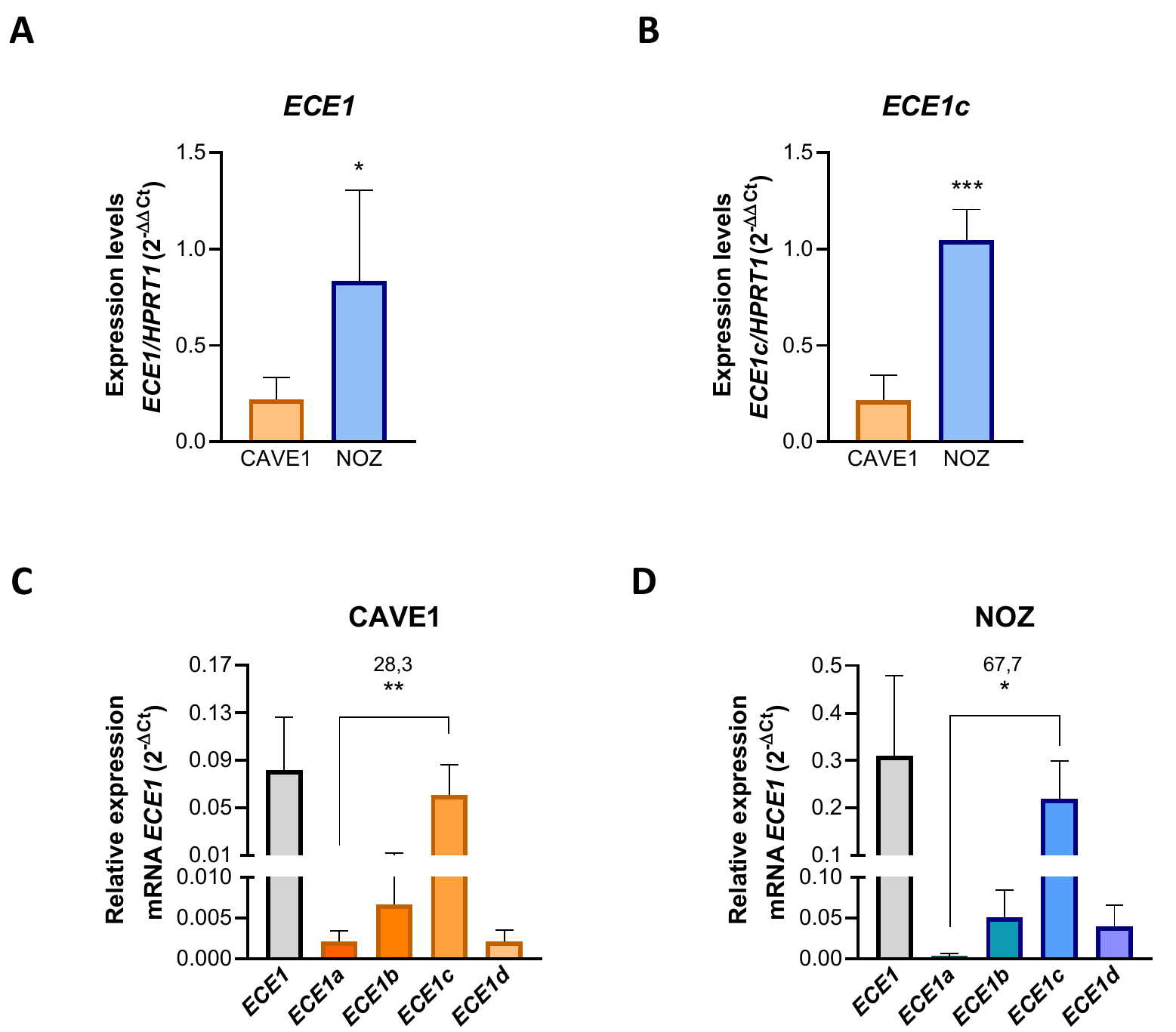

Supplement: Supplementary file 2 — Supplementary Material 2: Figure Supplementary 2. ECE1c is the predominant isoform in GBC cells. The expression profile of the ECE1 mRNA isoforms was determined by the 2−Δct method using the HPRT1 gene as the normalizer in both cell models and a common calibrator. The relative expression levels of total ECE1 (A) and ECE1c (B) were compared between both cell models. The relative expression levels of mRNA of the four isoforms (a-d) were compared in NOZ cells (C) and in CAVE1 cells (D). Statistical analysis was conducted using the Anova (*= p < 0.05, ** = p < 0.01; **** = p < 0.0001). [file 40659_2025_637_MOESM2_ESM.png]

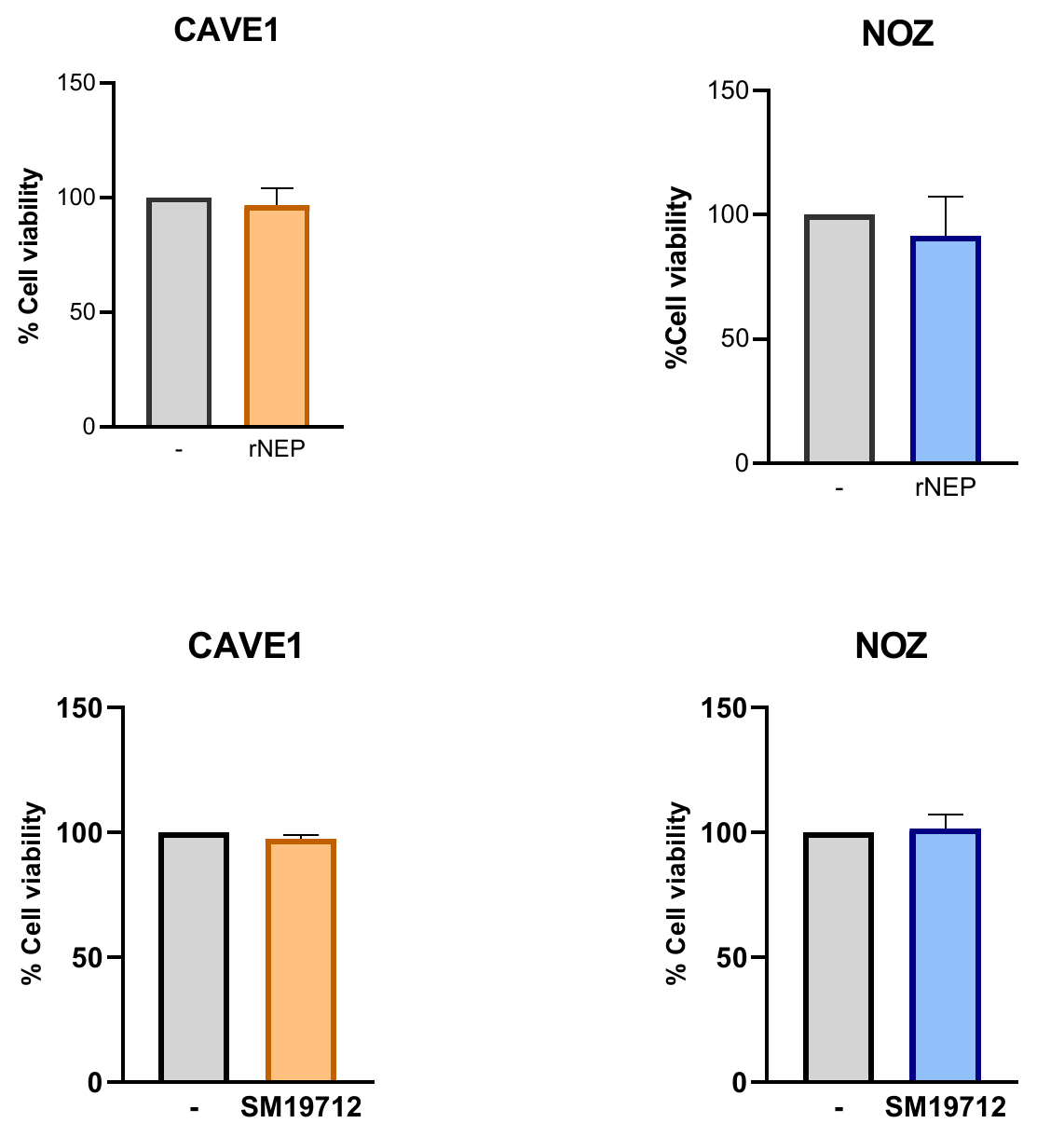

Supplement: Supplementary file 3 — Supplementary Material 3: Figure Supplementary 3. Cells were seeded in 96-well plates for 24h and treated with 0.2µg/ml rNEP or ECE1 inhibitor SM19712 (5 µM in NOZ and 20 µM in CAVE1) for 72h. Cells were incubated with MTS reagent for 2h and absorbance was measured at 490nm and plotted as percentage. Data represent averages ± SEMs (n = 3). ANOVA and Student’s tests were used. *p < 0.05 **p < 0.01. [file 40659_2025_637_MOESM3_ESM.png]

FIG.2A

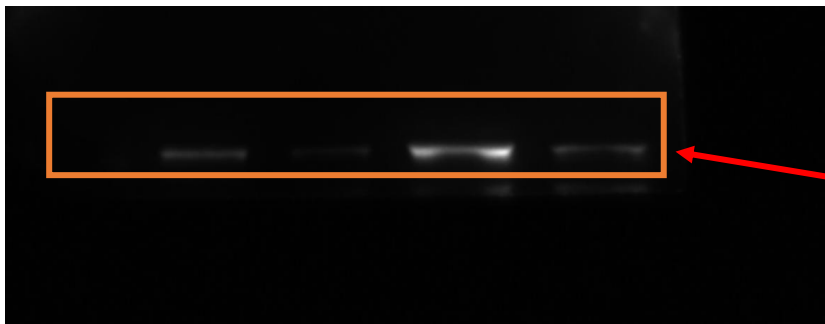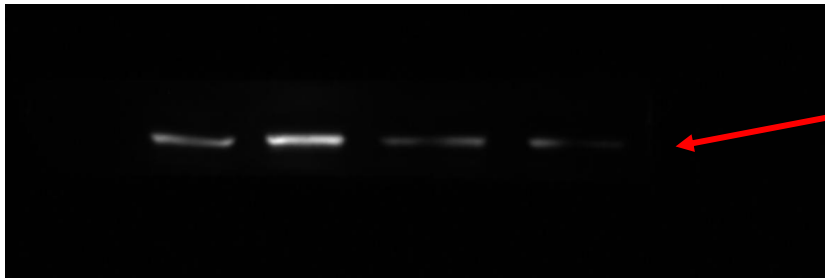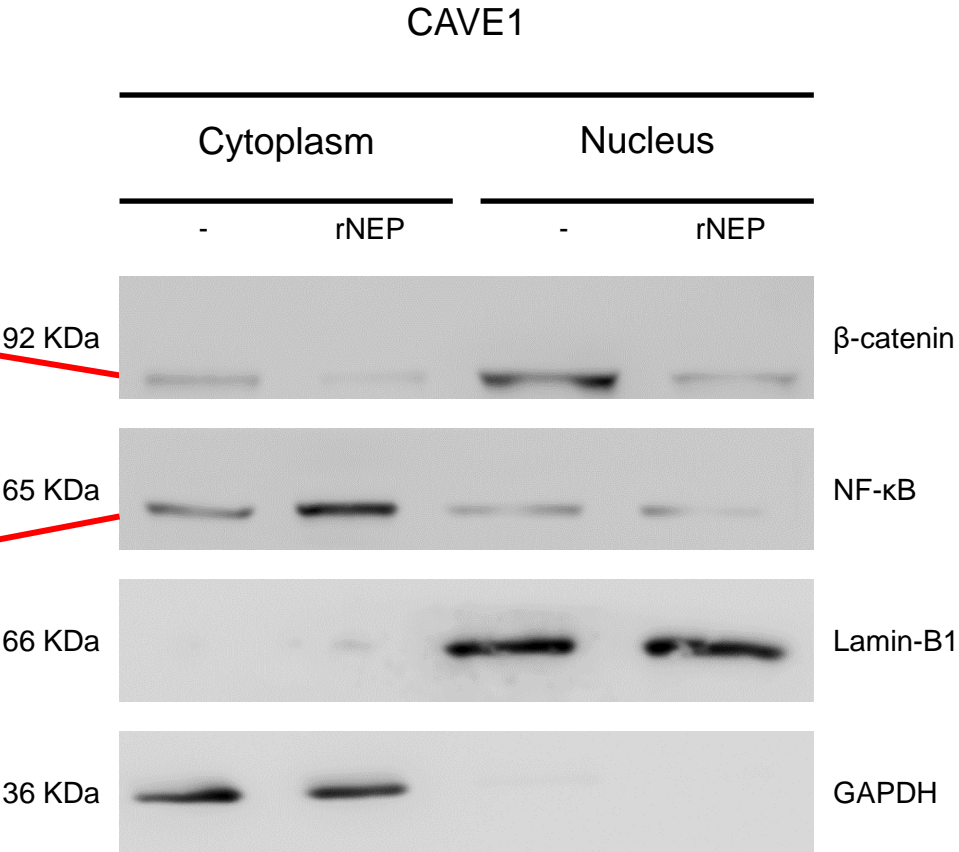

FIG.2A

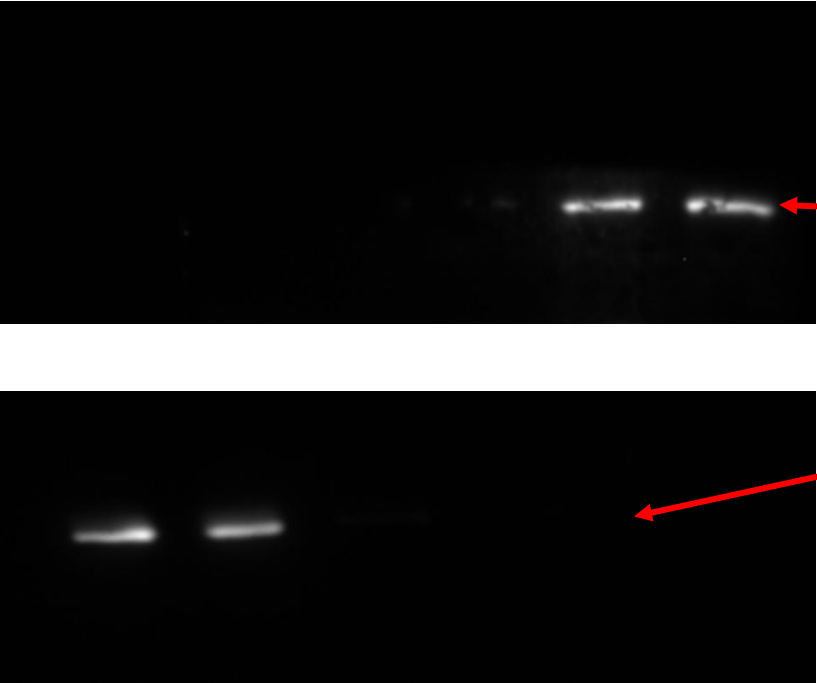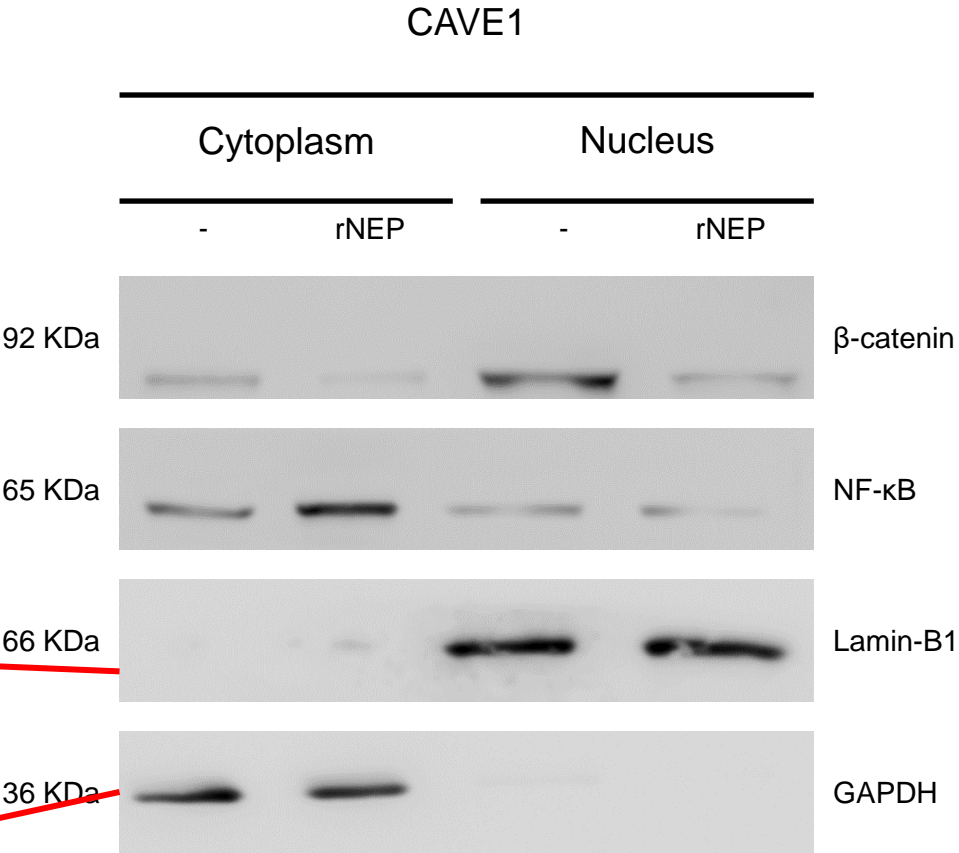

FIG.2A

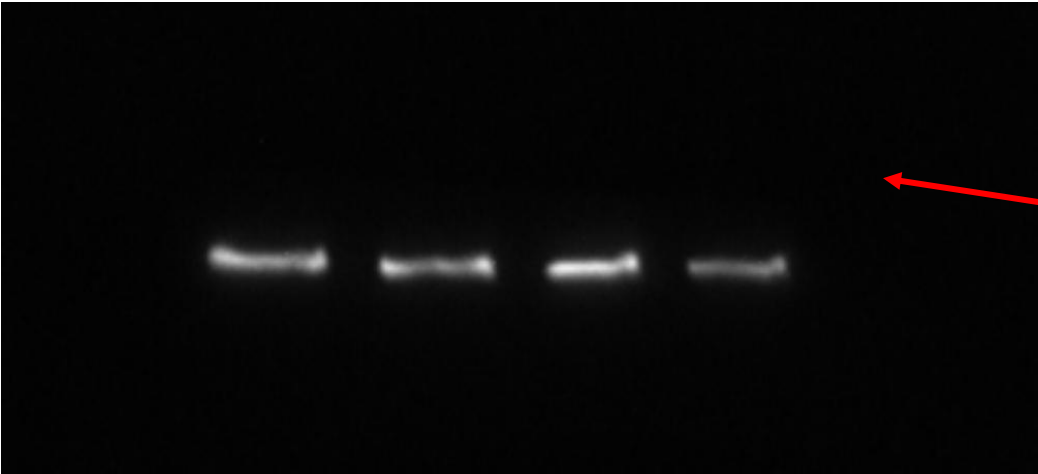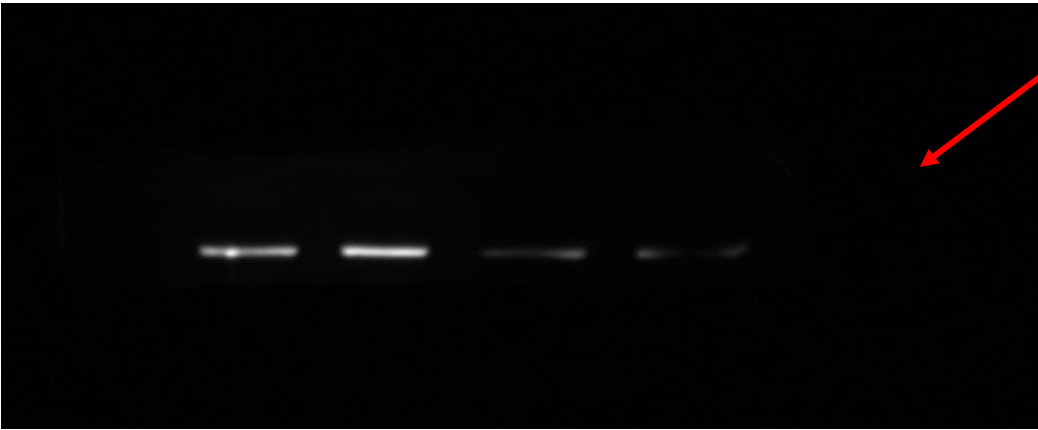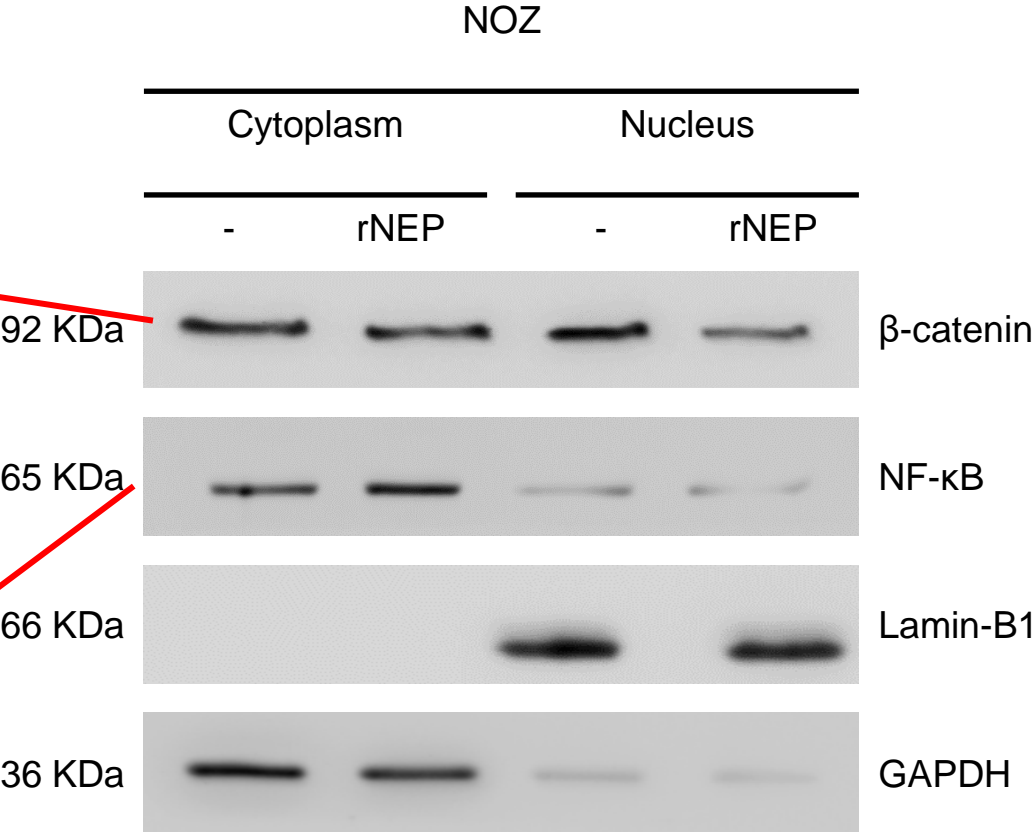

FIG.2A

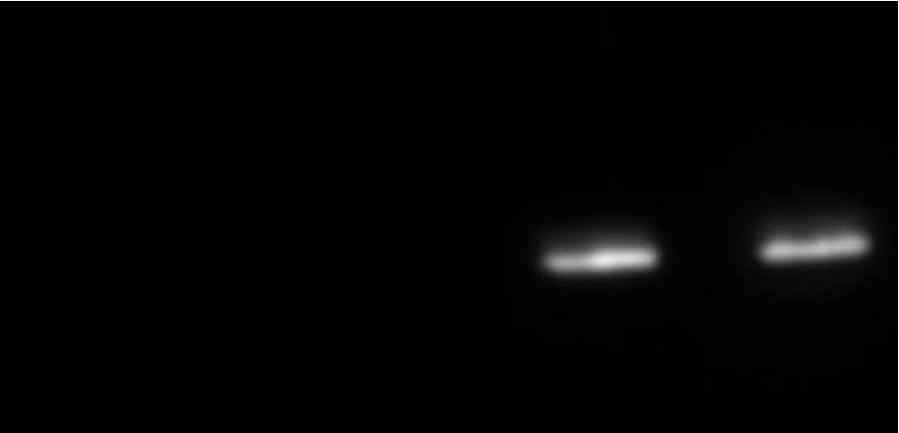

Lamin B1

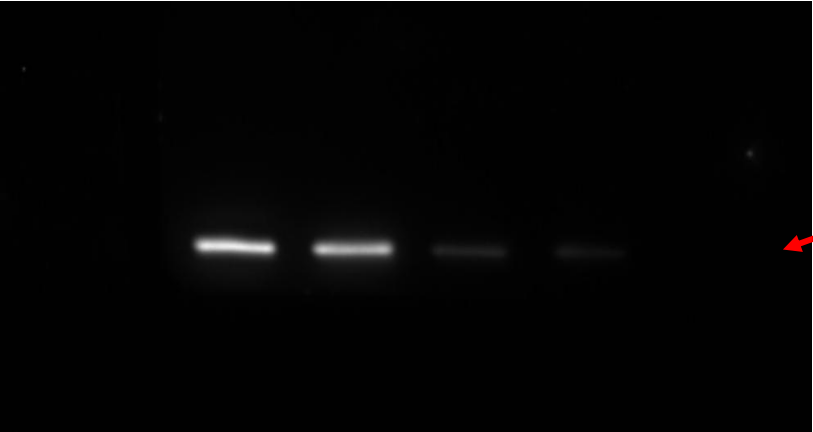

GAPDH

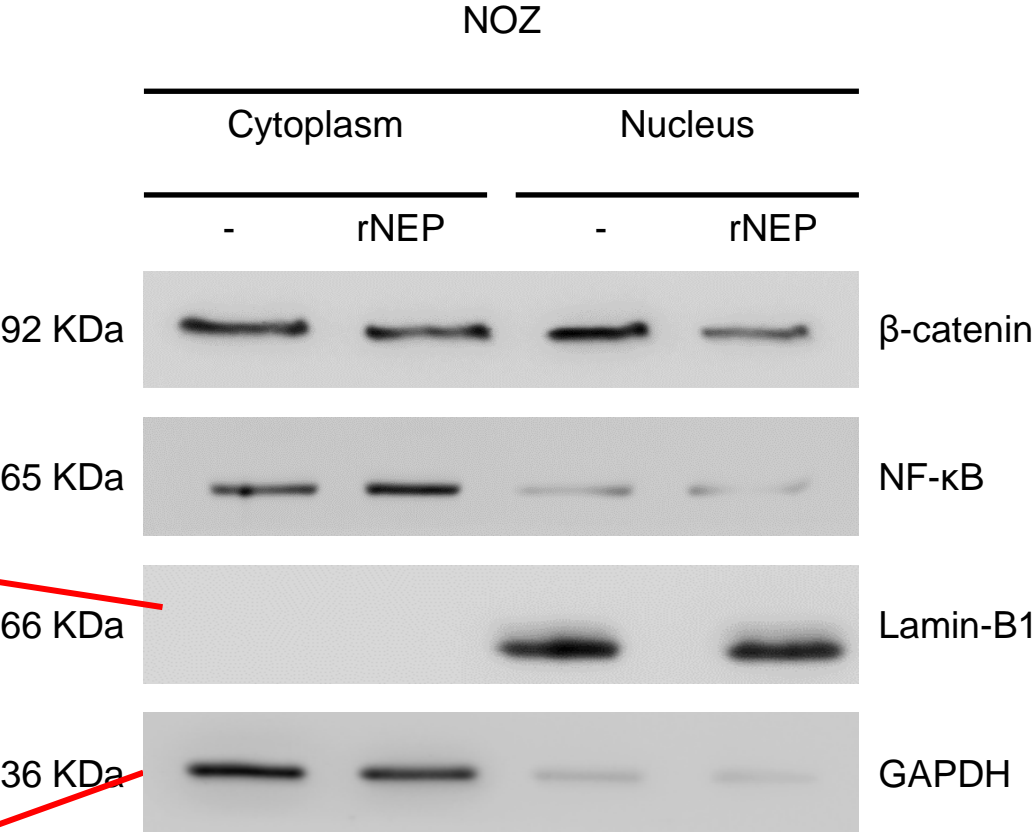

FIG.3A

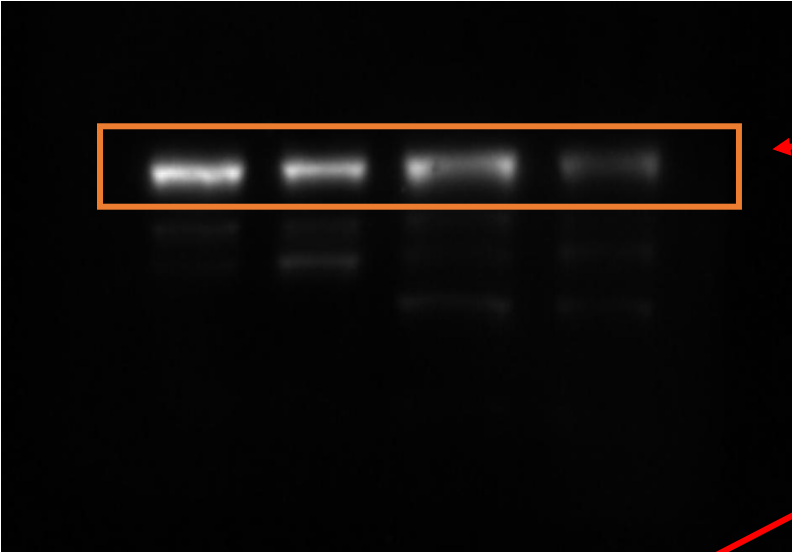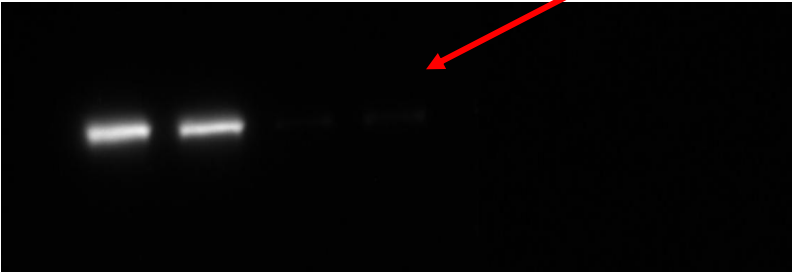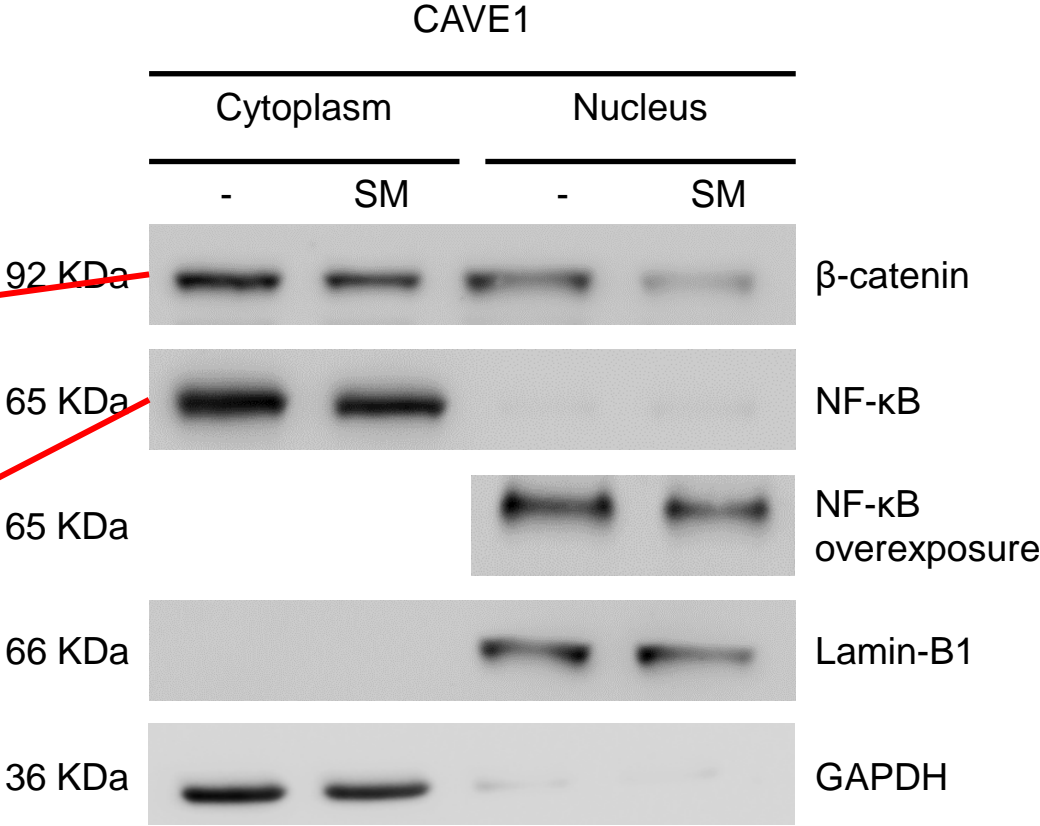

FIG.3A

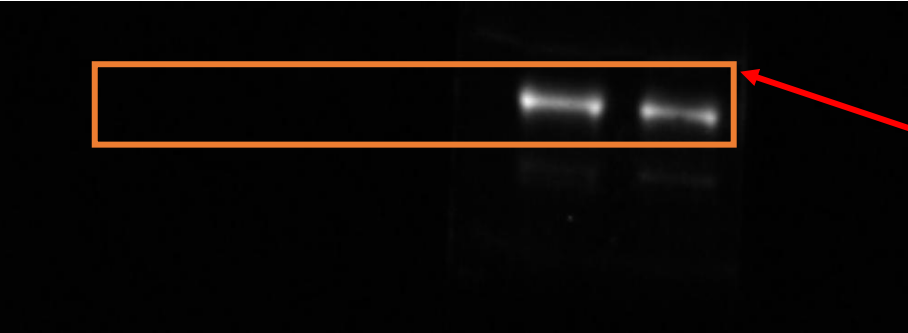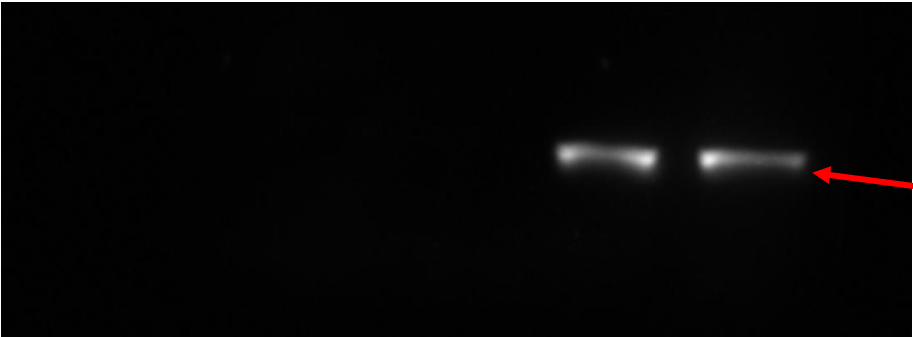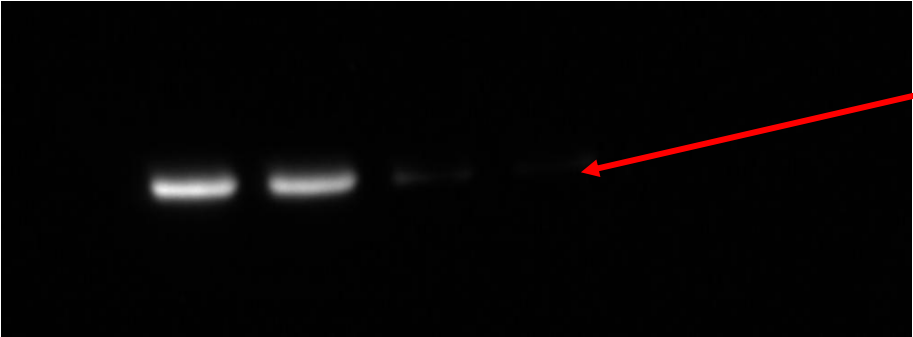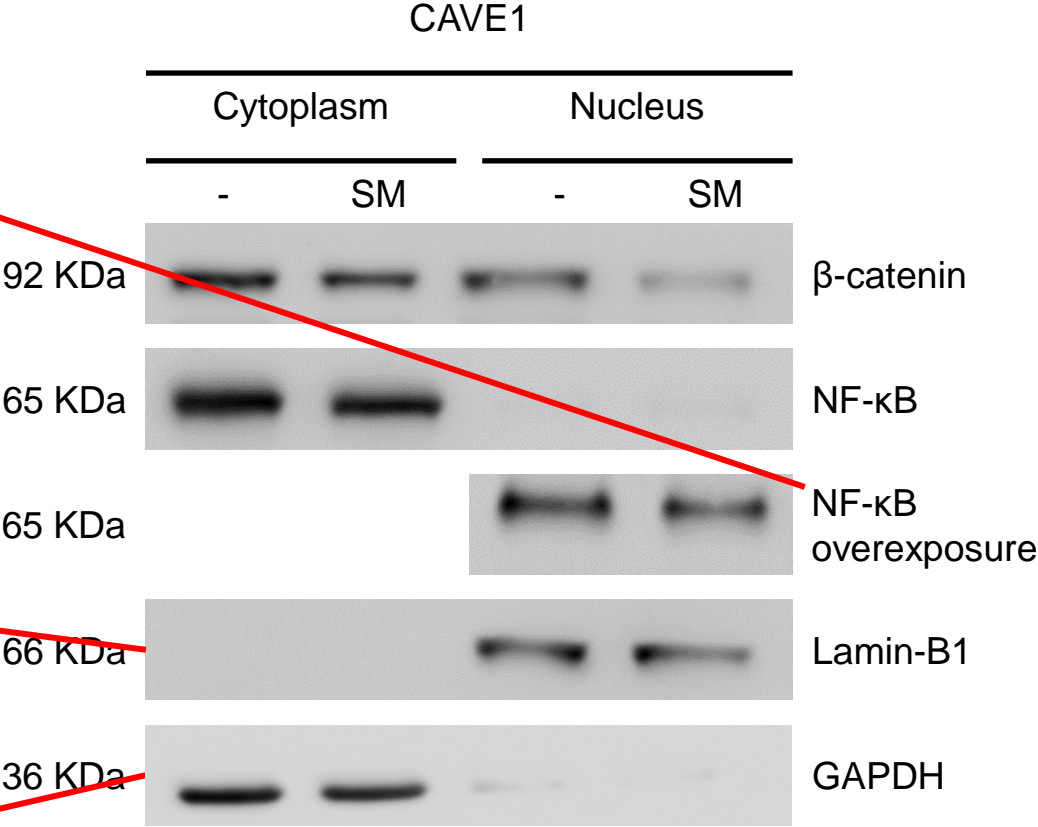

FIG.3A

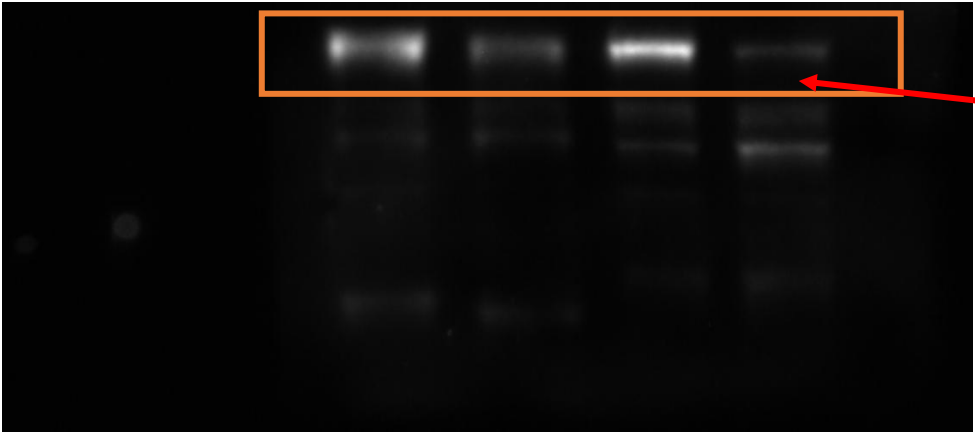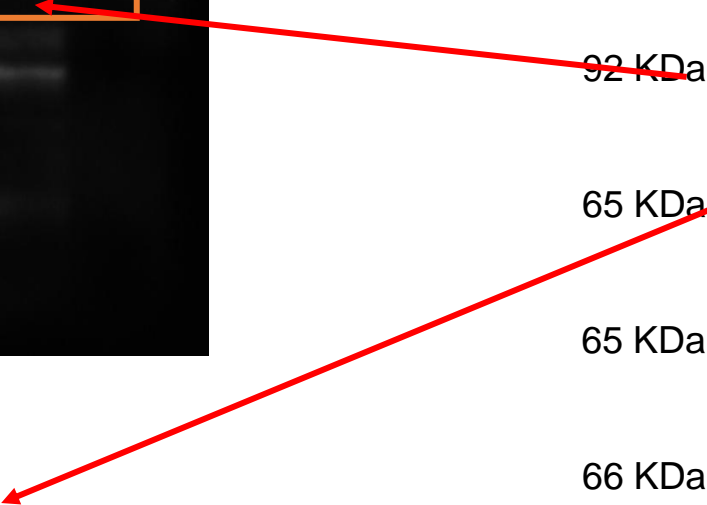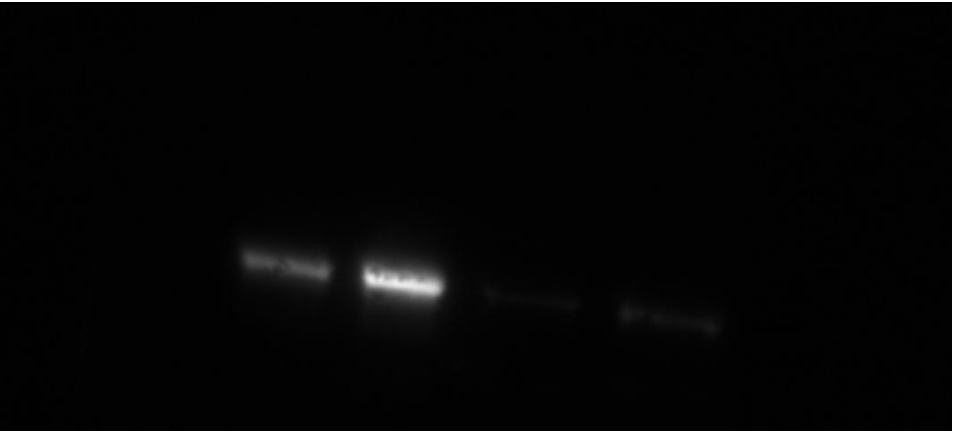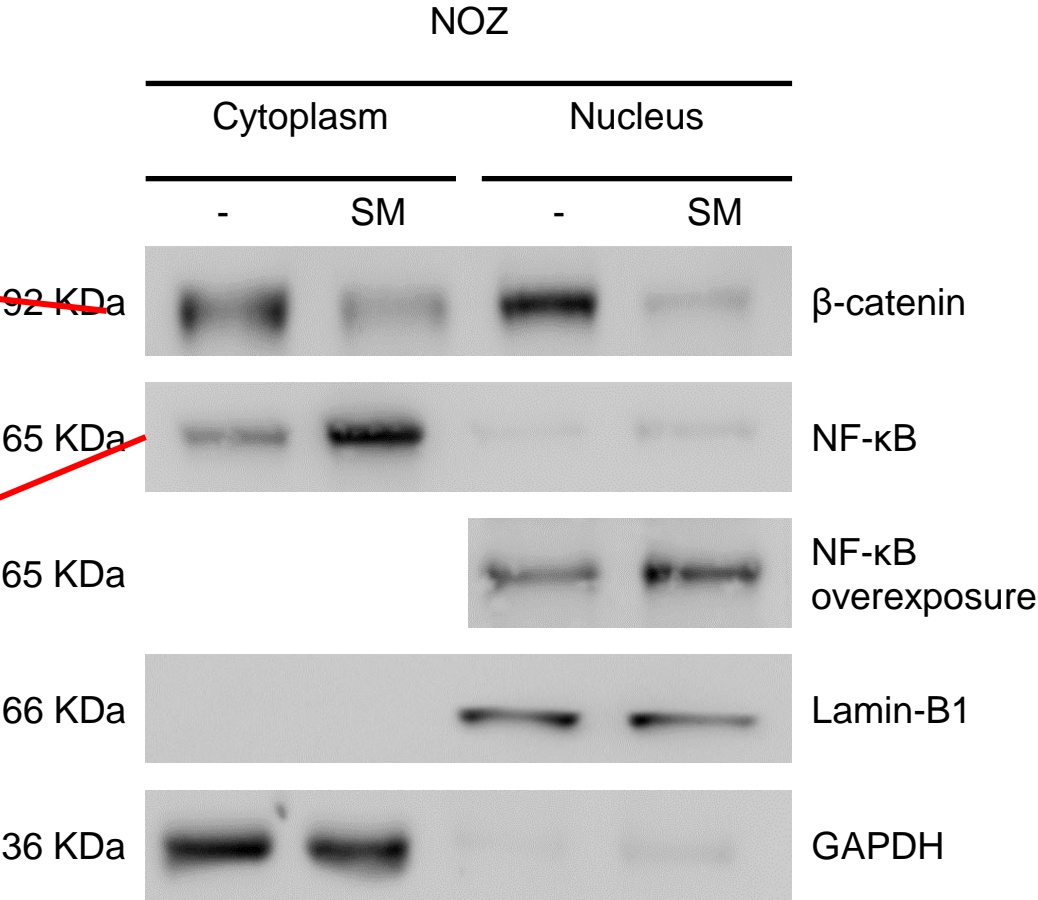

FIG.3A

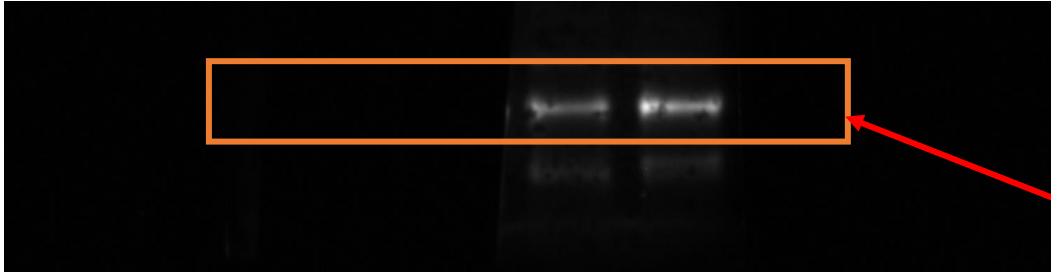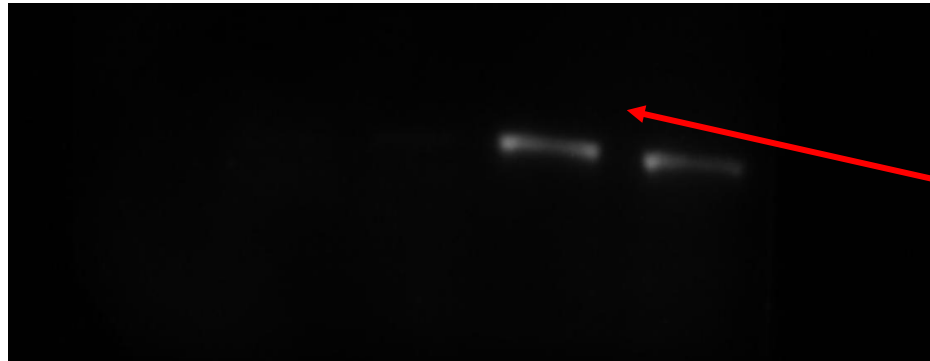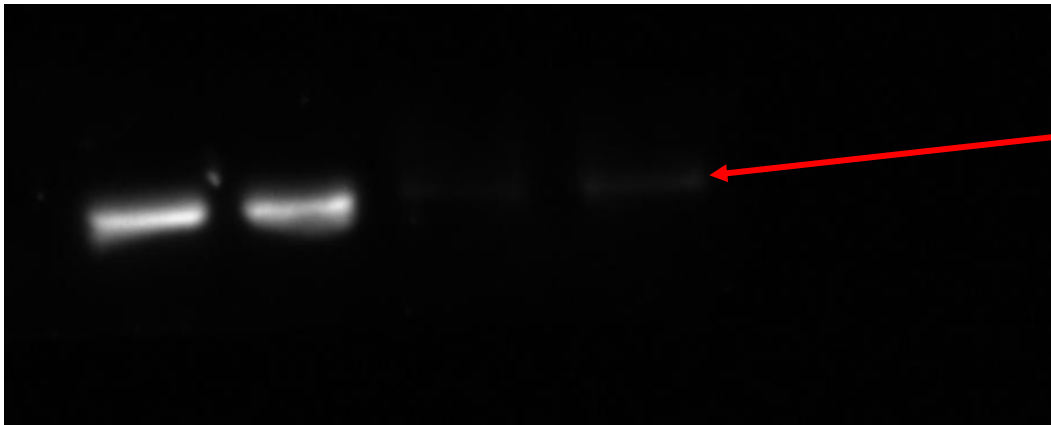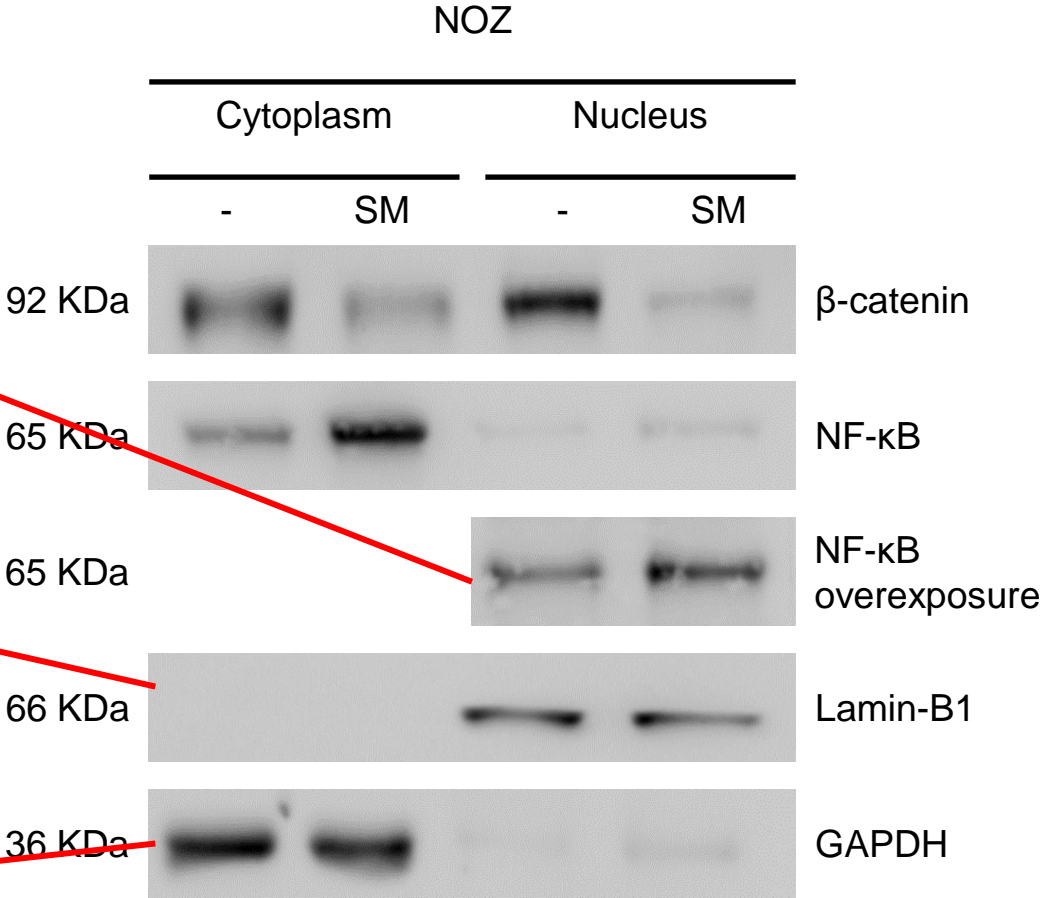

FIG.4A

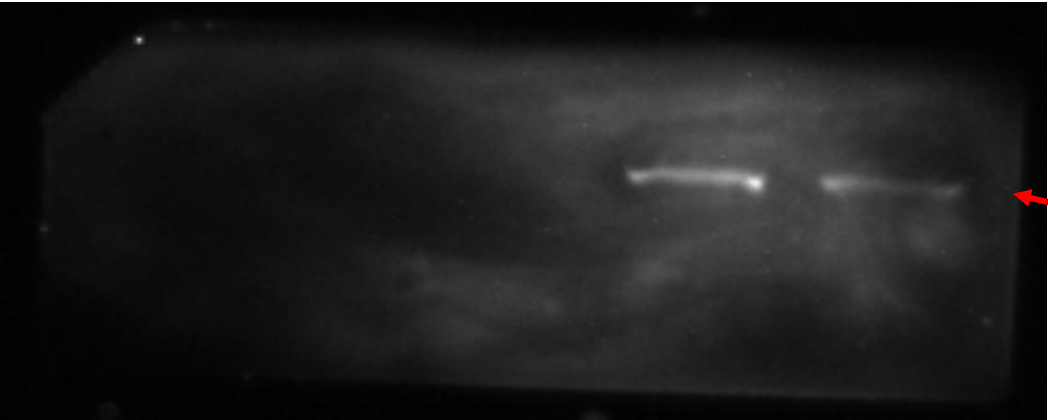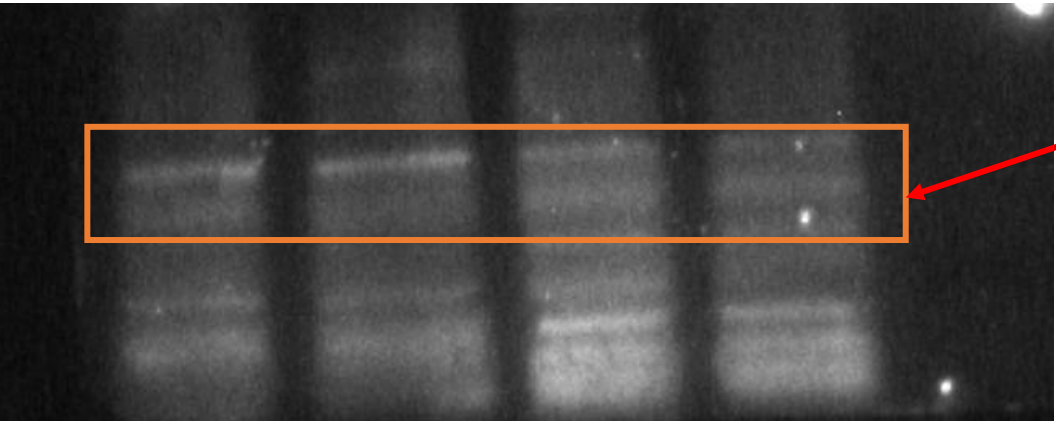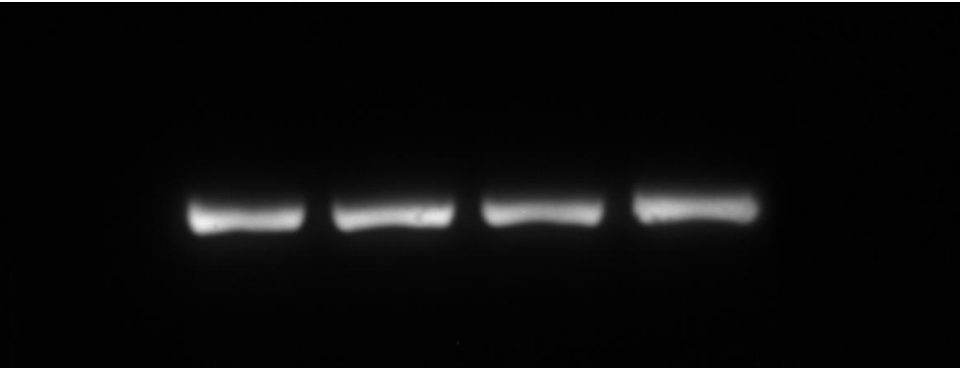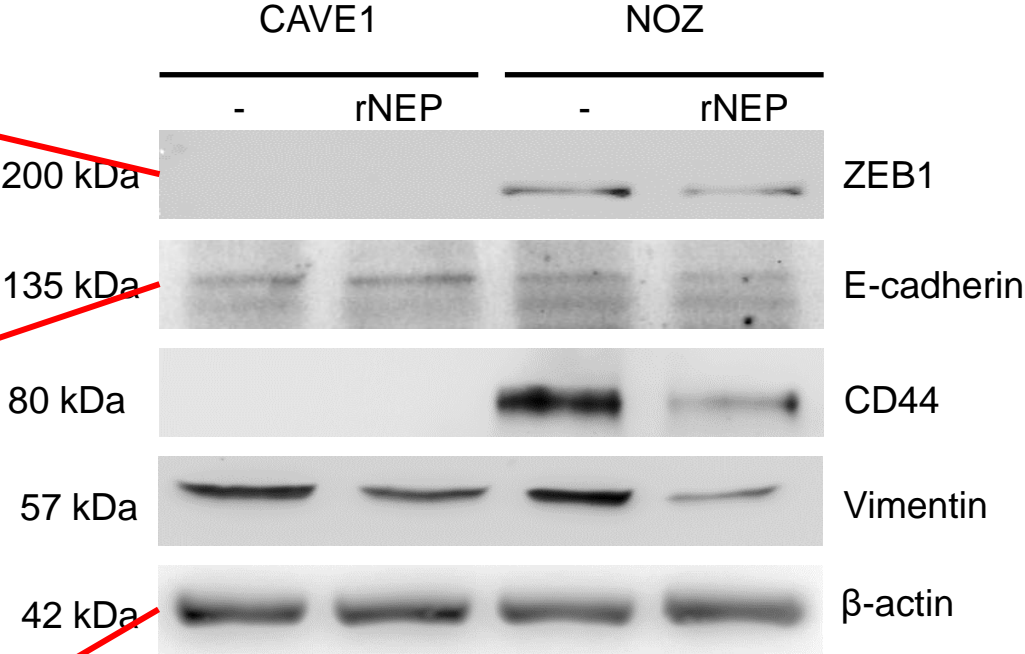

FIG.4 A

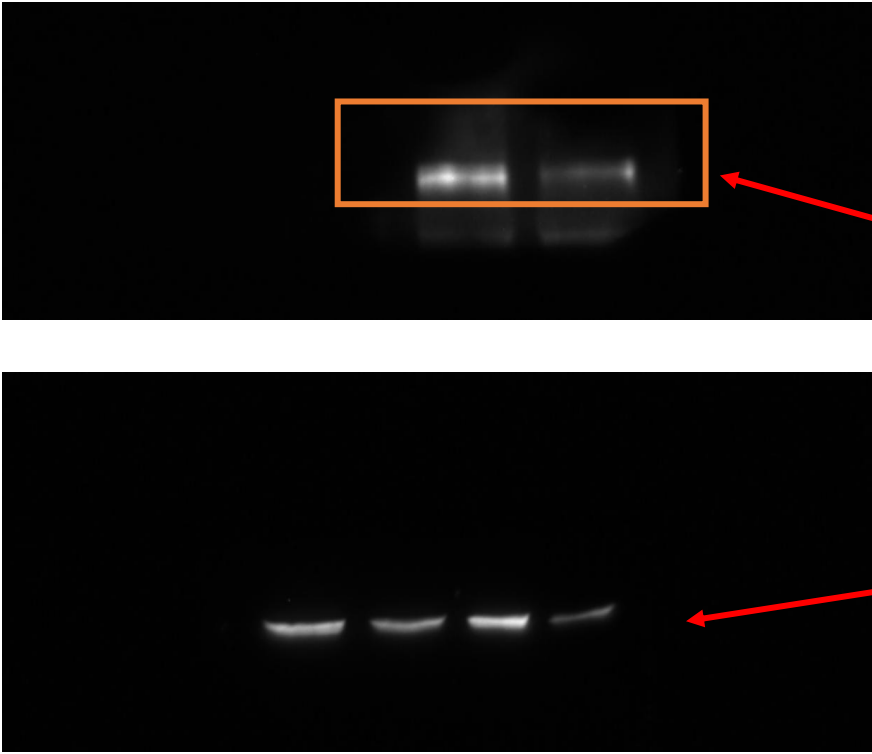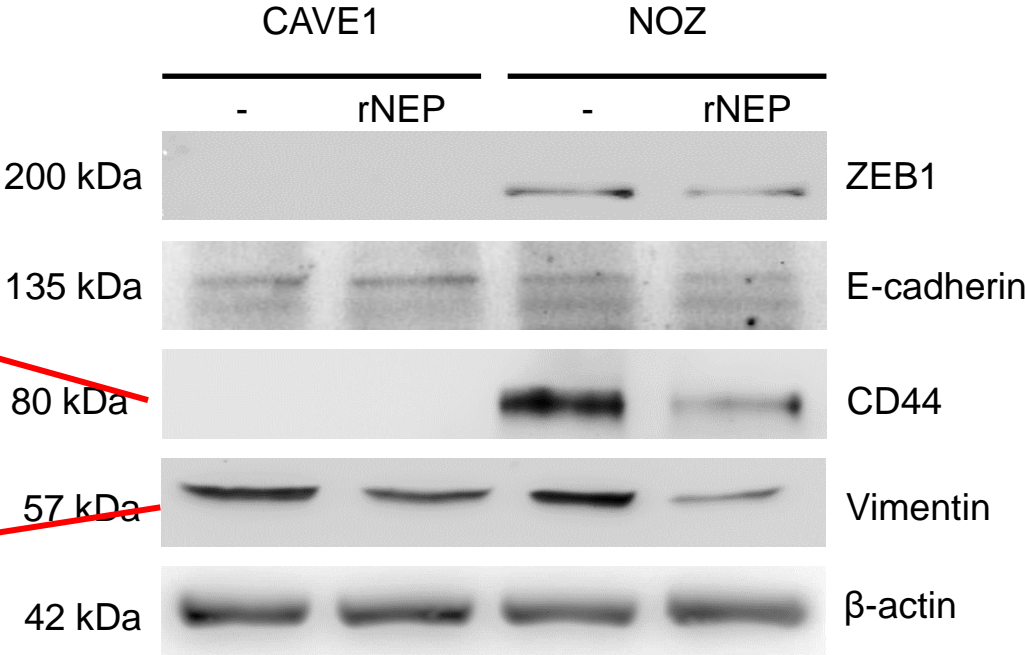

FIG.4D

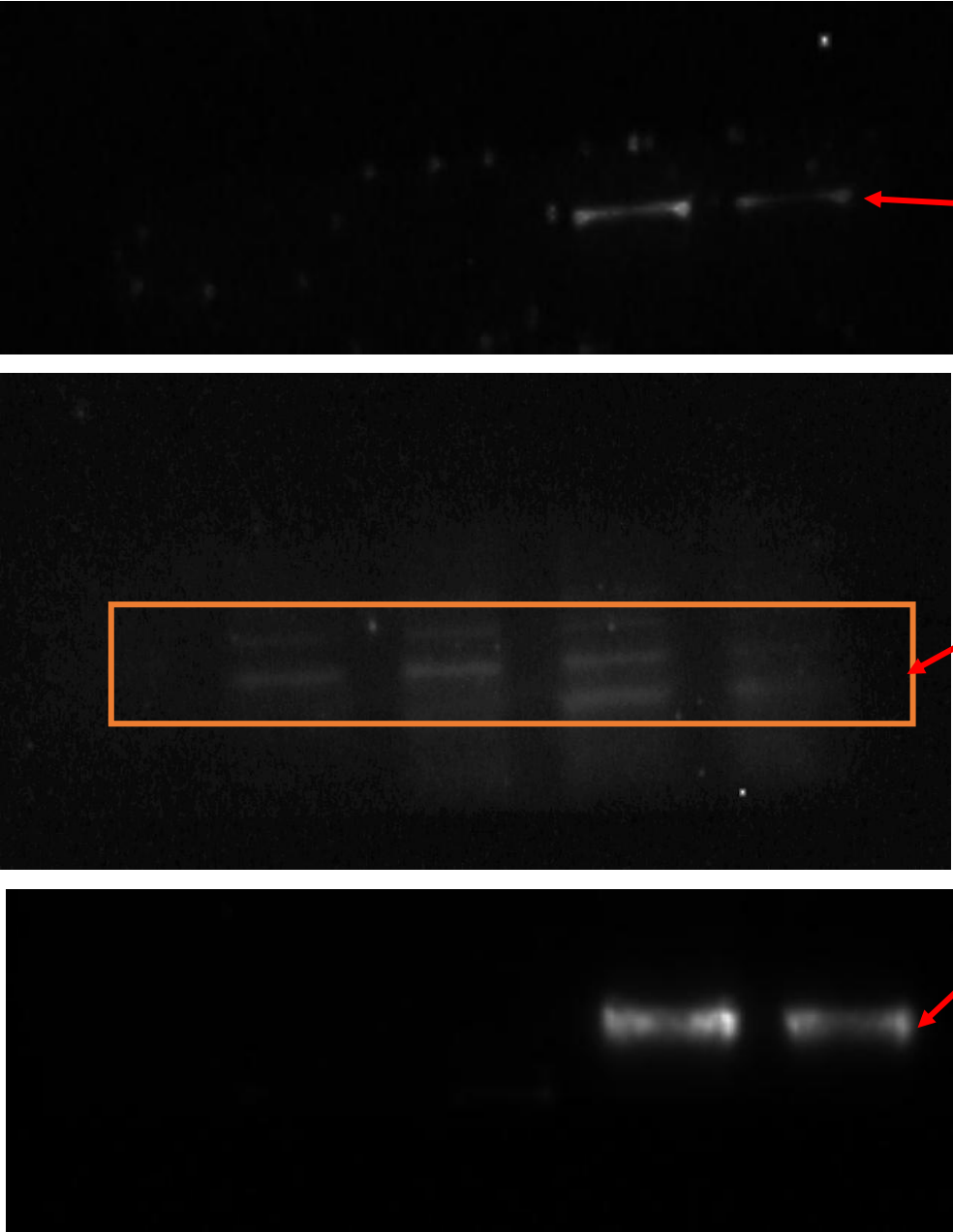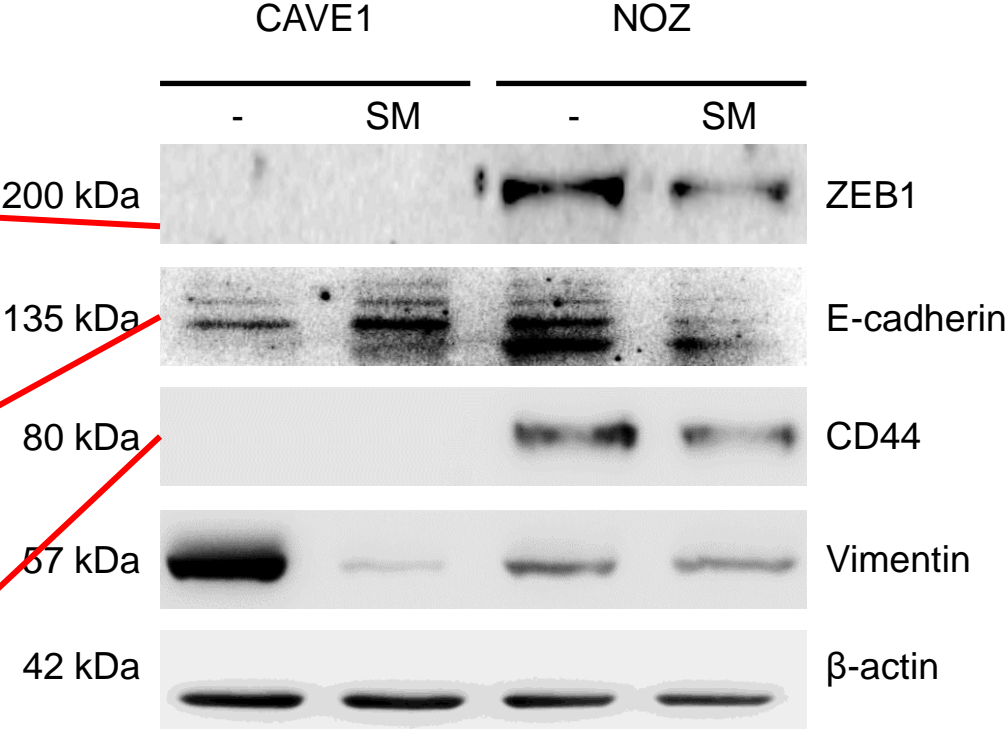

FIG.4D

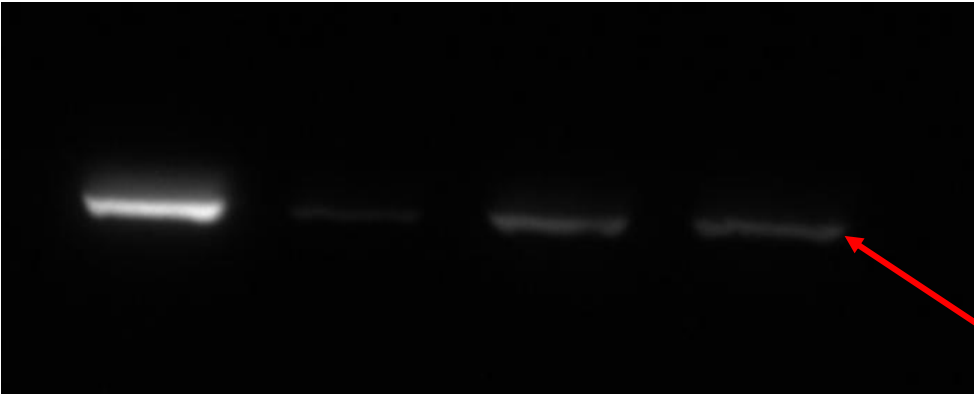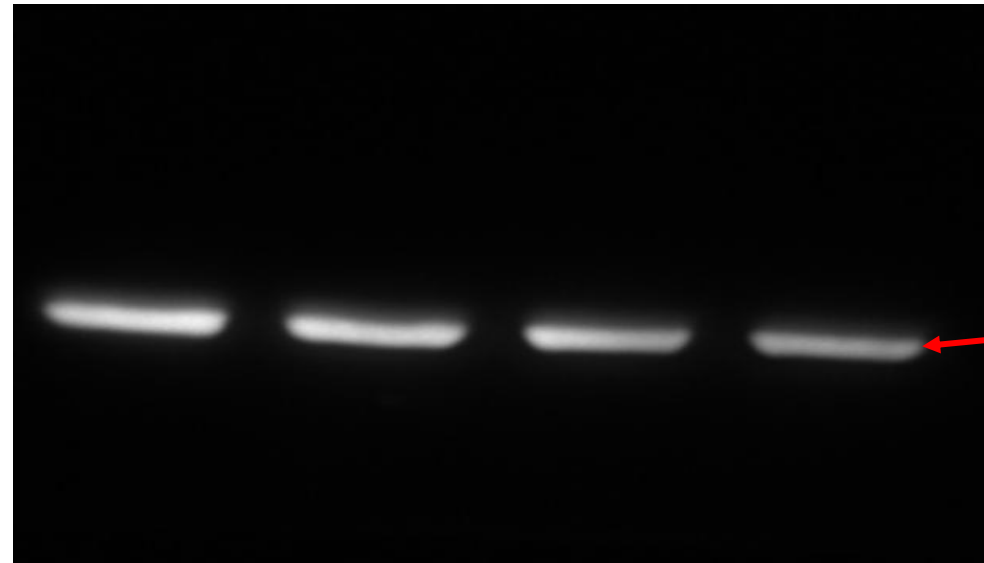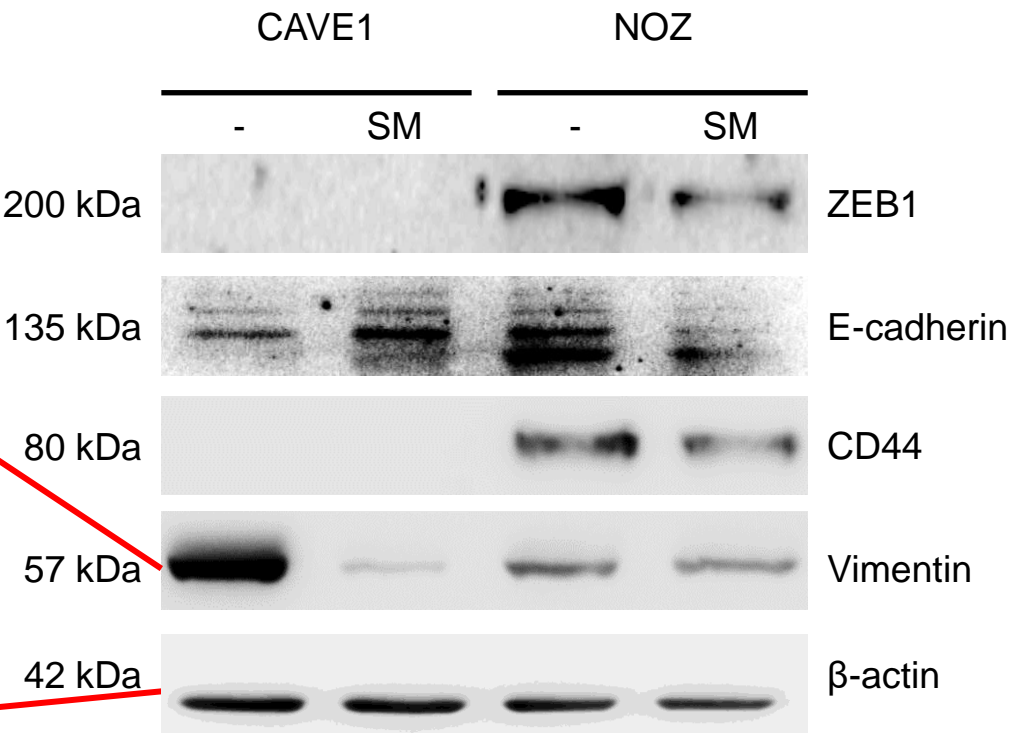

Supplement: Supplementary file 4 — Supplementary Material 4 [file 40659_2025_637_MOESM4_ESM.pdf]
